# Impact of Different Mass Drug Administration Strategies for Gaining and Sustaining Control of *Schistosoma mansoni* and *Schistosoma haematobium* Infection in Africa

**DOI:** 10.4269/ajtmh.19-0829

**Published:** 2020-05-12

**Authors:** Charles H. King, Nupur Kittur, Sue Binder, Carl H. Campbell, Eliézer K. N’Goran, Aboulaye Meite, Jürg Utzinger, Annette Olsen, Pascal Magnussen, Safari Kinung’hi, Alan Fenwick, Anna E. Phillips, Pedro H. Gazzinelli-Guimaraes, Neerav Dhanani, Josefo Ferro, Diana M. S. Karanja, Pauline N. M. Mwinzi, Susan P. Montgomery, Ryan E. Wiegand, William Evan Secor, Amina A. Hamidou, Amadou Garba, Daniel G. Colley

**Affiliations:** 1 Center for Global Health and Diseases, Case Western Reserve University, Cleveland, Ohio;; 2 Schistosomiasis Consortium for Operational Research and Evaluation, Center for Tropical and Emerging Global Diseases, University of Georgia, Athens, Georgia;; 3 Unité de Formation et de Recherche Biosciences, Université Félix Houphouët-Boigny, Abidjan, Côte d’Ivoire;; 4 Centre Suisse de Recherches Scientifiques en Côte d’Ivoire, Abidjan, Côte d’Ivoire;; 5 Programme National de Lutte Contre les Maladies Tropicales Négligées à Chimiothérapie Préventive (PNLMTN-CP), Abidjan, Côte d’Ivoire;; 6 Swiss Tropical and Public Health Institute, Basel, Switzerland;; 7 University of Basel, Basel, Switzerland;; 8 Section for Parasitology and Aquatic Pathobiology, Faculty of Health and Medical Sciences, University of Copenhagen, Copenhagen, Denmark;; 9 Centre for Medical Parasitology, Faculty of Health and Medical Sciences, University of Copenhagen, Copenhagen, Denmark;; 10 National Institute for Medical Research, Mwanza, Tanzania;; 11 Schistosomiasis Control Initiative, Imperial College, London, United Kingdom;; 12 Catholic University of Mozambique, Beira, Mozambique;; 13 Centre for Global Health Research, Kenya Medical Research Institute, Kisumu, Kenya;; 14 Centers for Disease Control and Prevention, Atlanta, Georgia;; 15 Réseau International Schistosomoses, Environnement, Aménagement et Lutte (RISEAL-Niger), Niamey, Niger;; 16 Department of Control of Neglected Tropical Diseases, Preventive Chemotherapy and Transmission Control Unit, World Health Organization, Geneva, Switzerland;; 17 Department of Microbiology, University of Georgia, Athens, Georgia

## Abstract

This report summarizes the design and outcomes of randomized controlled operational research trials performed by the Bill & Melinda Gates Foundation–funded Schistosomiasis Consortium for Operational Research and Evaluation (SCORE) from 2009 to 2019. Their goal was to define the effectiveness and test the limitations of current WHO-recommended schistosomiasis control protocols by performing large-scale pragmatic trials to compare the impact of different schedules and coverage regimens of praziquantel mass drug administration (MDA). Although there were limitations to study designs and performance, analysis of their primary outcomes confirmed that all tested regimens of praziquantel MDA significantly reduced local *Schistosoma* infection prevalence and intensity among school-age children. Secondary analysis suggested that outcomes in locations receiving four annual rounds of MDA were better than those in communities that had treatment holiday years, in which no praziquantel MDA was given. Statistical significance of differences was obscured by a wider-than-expected variation in community-level responses to MDA, defining a persistent hot spot obstacle to MDA success. No MDA schedule led to elimination of infection, even in those communities that started at low prevalence of infection, and it is likely that programs aiming for elimination of transmission will need to add supplemental interventions (e.g., snail control, improvement in water, sanitation and hygiene, and behavior change interventions) to achieve that next stage of control. Recommendations for future implementation research, including exploration of the value of earlier program impact assessment combined with intensification of intervention in hot spot locations, are discussed.

## INTRODUCTION

In 2009, the Bill & Melinda Gates Foundation (BMGF) funded the Schistosomiasis Consortium for Operational Research and Evaluation (SCORE), based at the University of Georgia. The seven most extensive projects at the core of SCORE’s operational research agenda involved large-scale, randomized comparison trials of different approaches to mass drug administration (MDA) using praziquantel for control of either *Schistosoma mansoni* or of *Schistosoma haematobium* in endemic areas of sub-Saharan Africa.^1^ In aiming to provide program managers and policy makers with better evidence for decision-making, SCORE’s research objective for these studies was to generate data on the relative effectiveness of community-wide treatment (CWT) versus school-based treatment (SBT)^2,3^ and on the relative effectiveness of annual praziquantel drug delivery as compared with schedules that involve holiday years—that is, years without praziquantel MDA.^4–8^ The focus on sub-Saharan Africa was based on this region having the greatest burden of schistosomiasis globally and the greatest need for information about optimal implementations at national and subnational scales.

After extensive consultation in 2009 with program managers, field trial researchers, biostatisticians, and representatives of the WHO, SCORE designed multiyear, large-scale cluster-randomized trials to study options for program implementation in areas having ≥ 10% *Schistosoma* infection prevalence among school-age children (SAC).^1^ At the time, WHO guidelines recommended every-other-year praziquantel treatment of all SAC in communities having SAC prevalence between 10% and 49%, and annual SAC treatment for communities having SAC prevalence ≥ 50%.^9^ For both categories, concurrent treatment of high-risk adults (i.e., those having regular contact with infested water, such as fishermen, farmers, irrigation workers, or women regularly coming in contact as part of their domestic tasks) was recommended, with the added proviso that in the areas of highest prevalence (≥ 50%), the entire community might need to be treated.^9^

In 2009, SCORE stakeholder partner deliberations agreed that these existing thresholds for implementation of mass treatment needed refinement and a better evidence base to establish the relative benefits of different age-group coverage formats and different schedules of targeted mass praziquantel delivery.^1^ In particular, it seemed quite unlikely that communities starting at a prevalence of 10% *Schistosoma* infection would have the same response to mass treatment as communities starting at a several fold higher prevalence (e.g., 49%), although they were classified in the same risk group by WHO guidelines.^9^ There was interest in finding out whether, when compared with SBT, using one or more years of CWT would have a greater impact on the prevalence and intensity of infection if, for example, CWT could achieve better coverage of SAC who did not attend school^2^ or better coverage of high-risk adults. There also was a lack of data as to whether every-other-year delivery of MDA could be “good enough” for morbidity control,^10^ that is, near elimination of heavy infections (defined as ≥ 400 *S. mansoni* eggs per gram of stool or ≥ 50 *S. haematobium* eggs per 10 mL of urine).^9^

As a result, two types of SCORE treatment trials were developed to focus separately on communities having starting prevalence 10–24%, designated as sustaining control studies, and on communities having starting prevalence ≥ 25%, designated as gaining control studies. Sustaining control studies in *S. mansoni* areas were conducted in Côte d’Ivoire and in western Kenya. Gaining control studies in *S. mansoni* areas were performed in Kenya and in Tanzania, and a gaining control study was performed in Mozambique in an area endemic for *S. haematobium*.^1^ Niger began both gaining and sustaining control studies in areas endemic for *S. haematobium.* However, these studies had then to be modified, as detailed in the following paragraphs.

This article summarizes the results of the SCORE gaining and sustaining studies and the redesigned Niger study. A separate report in this issue^11^ presents the results of separate SCORE studies that focused on “moving toward elimination” on Zanzibar and in *S. haematobium–*endemic areas of northern and central Côte d’Ivoire.^12^

## SCHISTOSOMIASIS CONSORTIUM FOR OPERATIONAL RESEARCH AND EVALUATION STUDY DESIGNS

**Figure 1.**
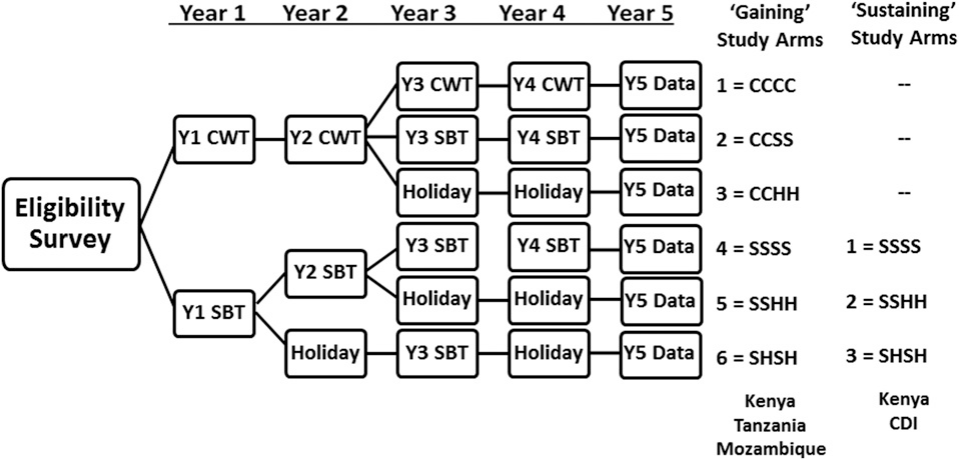
Study arms and timeline for the Schistosomiasis Consortium for Operational Research and Evaluation studies of gaining and sustaining control of schistosomiasis in sub-Saharan Africa. CDI = Côte d’Ivoire; CWT or C = community-wide treatment; H = drug holiday—a year when no praziquantel mass drug administration was provided; SBT or S = school-based treatment.

Development of harmonized protocols for the SCORE gaining and sustaining control studies has been described in detail in a previous publication.^1^ Briefly, it was decided that sustaining control studies (in locations with starting *Schistosoma* prevalence of 10–24%) would have community-level randomization to one of three different arms that compared different schedules of SBT with praziquantel. The higher prevalence gaining control areas were believed to need more aggressive intervention, but there was considerable uncertainty (and therefore equipoise) about whether more aggressive population coverage or more frequent drug delivery would yield better outcomes in terms of reducing prevalence and intensity of *Schistosoma* infection.^1^ Therefore, communities in the gaining control studies (starting prevalence ≥ 25%) were randomized to one of six arms: the three SBT schedules used in the sustaining control studies, plus three additional arms that included CWT. A diagram of the treatments given in the different study arms of the gaining and sustaining control studies is presented in Figure 1.

The studies were unblinded, community-level, cluster-randomized control trials, with communities receiving either CWT, SBT, or a CWT to SBT transition, with or without praziquantel holidays during the 4 years of trial. Although all school-age children were targeted for MDA, the chosen study endpoints were based on the evaluation of infection prevalence and intensity outcomes among 9–12-year-old children, the highest risk group, in each community in Year 5. Community eligibility was determined by initial preenrollment screening surveys for *S. mansoni* or *S. haematobium* infection among fifty 13–14-year-old schoolchildren. This screening age range was chosen for the community eligibility assessment because, ethically, it was felt that any child diagnosed as infected with schistosomes deserved prompt individual treatment and that pre-study treatment of a small number of 13–14-year-old individuals would not influence the underlying community baseline status among the trial’s sentinel 9–12-year-old age-group. In the screening phase, *S. mansoni* infection was detected by duplicate Kato–Katz thick smears^13^ taken from a single daily stool and *S. haematobium* infection was diagnosed by the detection of microhematuria by urine reagent strip testing.^14,15^

### Sustaining control studies.

These cluster-randomized studies involved comparison of three different regimens of SBT of SAC in medium prevalence communities. The harmonized criteria for SCORE studies on sustaining control (see protocol in Supplemental Appendix A for details) were as follows:

1. Community inclusion criteria
  - *Schistosoma* infection prevalence of 10–24% (by egg detection) among children aged 13–14 years in eligibility surveys before the start of the study, or, for *S. haematobium* studies, a 5–20% prevalence of microhematuria by reagent strip testing. The option for microhematuria-based surveys for village eligibility was based on their relative ease of performance and the rapidity of their results. The lower 5–20% cutoffs for *S. haematobium* prevalence estimation by dipstick testing (versus egg count prevalence) were chosen based on the past experience of SCORE investigators working in Kenya, Zanzibar, and Niger, who had established that only about 50–60% of light and very light infections found by egg filtration have detectable dipstick hematuria.^16,17^ For the preliminary village eligibility surveys, but not the SCORE studies themselves, 5–20% dipstick-positive prevalence among 13–14 year olds was taken to indicate a moderate prevalence community, where *S. haematobium* egg count prevalence was likely to be in the 10–24% range.
  - Presence of a community primary school with at least 100 children aged 9–12 years who were available for surveillance. Adjacent communities could be combined as an implementation unit to make up this number of children to treat and monitor.
  - Community-level assent, and willingness of parents/guardians to provide written informed consent for individual children’s participation. Children’s assent for testing and praziquantel treatment.
2. Community exclusion criteria
  - Communities having baseline prevalence outside the 10–24% range.
  - High rates (> 10%) of mixed *S. mansoni* and *S. haematobium* infection.
3. The primary outcomes of the study were the prevalence and intensity of infection among a random sample of 100 local SAC, aged 9–12 years in Year 5, after 4 years of implementation of the community’s assigned treatment schedule.
4. As shown in Figure 1, the three study arms for comparison were as follows: Arm 1, annual SBT for 4 years; Arm 2, annual SBT for 2 years, followed by 2 years without praziquantel treatment (praziquantel holiday years); and Arm 3, two rounds of SBT given every other year, starting in Year 1, with praziquantel holidays in the intervening years (years 2 and 4).
5. Primary statistical analysis focused on comparing the WHO-recommended intervention, that is, every-other-year MDA,^9,18^ to more aggressive annual treatment, or to the impact of two annual treatments followed by a 2-year pause.
6. Data collected for secondary analyses were focused on community-level factors related to treatment response and the relative costs involved in implementing each study arm. These factors included information on local water and sanitation features, interval rainfall and drought, and the treatment coverage achieved among SAC in each community. In addition to monitoring 9–12-year-old children, the prevalence and intensity of infection among 100 first-year schoolchildren (aged 5–8 years) was assessed in the first and fifth year of each study as an indirect gage of recent rates of local transmission.

### Gaining control studies.

These cluster-randomized studies involved comparison of six different regimens of CWT and/or SBT in communities with baseline infection prevalence ≥ 25%. The harmonized criteria for SCORE studies on gaining control were as follows:

1. Community inclusion criteria
  - *Schistosoma* infection prevalence of ≥ 25% (by egg detection) among children aged 13–14 years in eligibility surveys performed before the start of the study, or, for *S. haematobium* studies, a ≥ 21% prevalence of microhematuria on reagent strip testing.
  - School size and participation criteria as for sustaining control studies, described earlier.
2. Community exclusion criteria
  - Communities having baseline prevalence below 25%
  - High rates (> 10%) of mixed *S. mansoni* and *S. haematobium* infection.
3. As in the sustaining control studies, the primary study outcomes were the prevalence and intensity of infection among a random sample of local 9–12-year-old children in Year 5, after 4 years of implementation of the community’s assigned treatment schedule.
4. As shown in Figure 1, the six study arms included in gaining control studies were as follows: Arm 1, CWT annually for 4 years; Arm 2, CWT annually for 2 years followed by SBT for 2 years; Arm 3, CWT for 2 years followed by two praziquantel holiday years; and Arms 4–6 were identical to Arms 1–3 of the sustaining control studies, described earlier.
5. Primary statistical analysis focused on comparing the WHO standard of care (SBT every year, given when baseline SAC prevalence is ≥ 50%) or every-other-year MDA (when baseline prevalence is 10–49%)^9,18^ to more aggressive CWT for 2 or 4 years, or to CWT tapering to SBT over 4 years.
6. As was performed for the sustaining control studies, data collected for secondary analyses included assessment of community-level factors related to treatment response, treatment coverage, and the relative costs involved in implementing each study arm. The prevalence and intensity of infection were also assessed in 100 previously untreated 5–8-year-old first-year students in each community as an indicator of ongoing transmission. For these gaining control studies, 50 adults aged 20–55 years in each community were also examined in years 1 and 5 both to gage the influence of baseline adult infection status on local response to treatment intervention and assess the direct or indirect impact of 4 years of local MDA implementation on the adult levels of infection in the final year (Year 5).

Power analysis performed during development of the harmonized protocols indicated that the inclusion of 25 communities in each study arm, with monitoring of 100 children per community, would have 90% power to detect a significant (two-tailed α = 0.05) between-arm difference of 11% and 9% in the Year 5 outcomes for gaining and sustaining studies, respectively. The assumed overdispersion factor was ϕ = 5.0, and the expected prevalence shift was from an average 50% prevalence in Year 1, to 15% prevalence in Year 5 in gaining control studies and from 24% to 10% in sustaining control studies.^1^ The final selection of 25 communities per arm per study was based on these projections and also the practical limitations (program capacity for implementation) of proposed partner sites in endemic countries.

Additional operational research on morbidity control was layered onto these gaining control studies. Cohort morbidity studies on the impact of treatment on early childhood morbidity were embedded in 8–12 communities in each of the *S. haematobium* and *S. mansoni* gaining control studies.^10^ Their results are described in further detail in a separate article in this issue.^19^ A subset of communities in the gaining control studies were also used for the companion studies of intermediate host snail abundance and infection^20^ and the influence of MDA on population genetics of the respective *Schistosoma* parasites.^21^

### Midpoint structural reorganization of the Niger gaining and sustaining control studies.

In the gaining and sustaining control studies in Niger, there was a significant protocol deviation with respect to randomization of study communities, in which geographically clustered groups of 25 communities were randomly assigned to each of the study arms rather than having the different community interventions distributed randomly.

**Figure 2.**
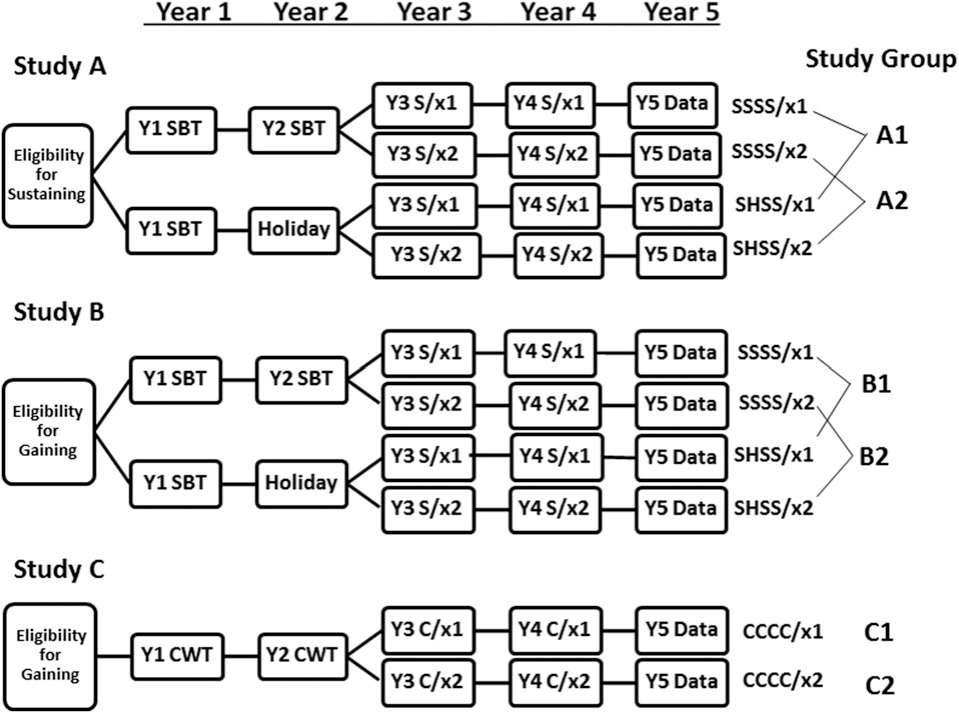
Revised study design for Schistosomiasis Consortium for Operational Research and Evaluation’s Niger *Schistosoma haematobium* control studies. CWT or C = community-wide treatment; H = drug holiday—a year when no praziquantel mass drug administration (MDA) was provided; SBT or S = school-based treatment; x1 = one MDA per year in years 3 and 4; x2 = two MDAs per year in years 3 and 4.

Subsequently, the Niger protocols were modified after Year 2. The new objective was to compare twice-yearly treatment to once-yearly treatment. In Year 3, all study arms for both studies were divided into two parts, with half of the communities in each category randomized to receive subsequent twice-a-year (biannual) MDA, whereas the other half received annual MDA (see Figure 2). This created three distinct randomized comparison sub-studies. Sub-study A was derived from communities initially enrolled in the Niger sustaining control study, and sub-studies B and C were derived from communities initially enrolled in the gaining control study. The sub-studies A, B, and C were analyzed separately.

1. Primary outcomes of the study were the prevalence and intensity of *Schistosoma* infection among a random sample of local SAC, aged 9–12 years, in Year 5, after 4 years of implementation of the community’s assigned treatment schedule.
2. Data collected for secondary analysis was the same as for the other studies of gaining and sustaining control. As in those studies, the prevalence and intensity of infection was additionally assessed in 100 first-year students in years 1 and 5. In those communities that were initially part of the gaining control study, data were also collected on 50 adults aged 20–55 years in years 1 and 5.

## KEY FINDINGS FROM THE GAINING AND SUSTAINING CONTROL STUDIES

**Figure 3.**
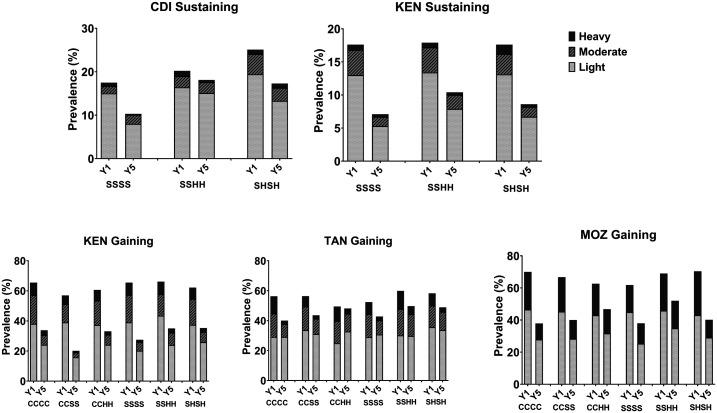
Overall prevalence, by infection categories of light, moderate, and heavy intensity categories at Year 1 (baseline; Y1) and Year 5 (study endpoint; Y5) in the Schistosomiasis Consortium for Operational Research and Evaluation gaining and sustaining control studies. The study arms indicated are detailed in Figure 1. CDI = Côte d’Ivoire; C = community-wide treatment; H = drug holiday—a year when no praziquantel mass drug administration was provided; KEN = Kenya; MOZ = Mozambique; S = school-based treatment; TAN = Tanzania.

In aggregate, the large-scale SCORE gaining and sustaining studies provided important new evidence on programmatically critical issues relating to how best to gain and sustain the morbidity control of schistosomiasis. Mass drug administration with praziquantel reduced community-level prevalence and the mean intensity of *Schistosoma* infection across all study arms and in all SCORE study countries (AE Phillips, et al., personal communication and M Ouattara et al., personal communication).^22–28^ Figure 3 shows the Year 1 (baseline) and Year 5 prevalence values by treatment study arm for the sustaining control studies for *S. mansoni* in Côte d’Ivoire and Kenya, the gaining control studies for *S. mansoni* in Kenya and Tanzania, and the gaining control study for *S. haematobium* in Mozambique. Prevalence by intensity of infection (light, moderate, or heavy)^9^ are also indicated in Figure 3. Arms 3 (CCHH) and 5 (SSHH) in gaining control studies and Arm 2 (SSHH) in sustaining control studies involved skipping two consecutive years of praziquantel MDA as drug holidays. When compared with arms that had four annual treatments, those arms with alternate or consecutive holiday years had smaller reductions in prevalence in Côte d’Ivoire, Tanzania, Mozambique, and Kenya (see Supplemental Tables S1–S5). However, because of wide variability in individual community responses to their assigned MDA program, this apparent disadvantage of scheduled holidays was not statistically significant overall.

**Figure 4.**
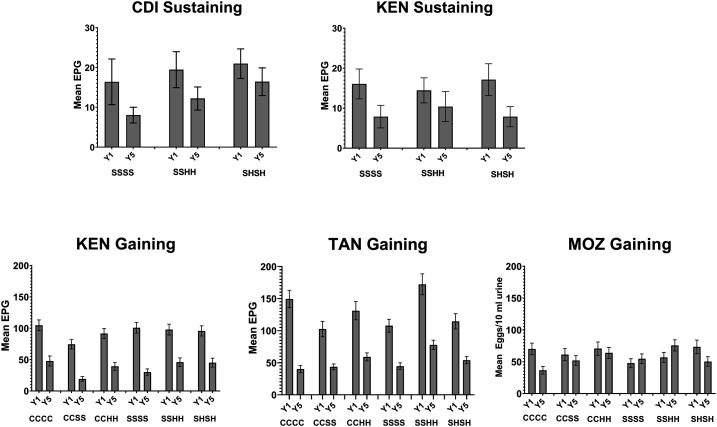
Mean community-level infection intensity in *Schistosoma mansoni* eggs per gram feces, or *S. haematobium* eggs per 10 mL urine at Year 1 (baseline; Y1) and Year 5 (study endpoint; Y5) in the Schistosomiasis Consortium for Operational Research and Evaluation gaining and sustaining control studies. The study arms indicated are detailed in Figure 1. CDI = Côte d’Ivoire; C = community-wide treatment; H = drug holiday—a year when no praziquantel mass drug administration was provided; KEN = Kenya; MOZ = Mozambique; S = school-based treatment; TAN = Tanzania.

Prevalence of moderate and heavy infections declined among 9–12-year-old children in all study arms for all gaining and sustaining control studies. Figure 4 demonstrates the change in community-level mean infection intensity from Year 1 to Year 5 in the various studies.

Although there were initial reductions in response to MDA implementation, which accord with projections of pre-study deterministic modeling,^29^ the trajectories of aggregate prevalence and intensity impact tended to plateau after 2–3 years in most settings.^30^ We observed some larger-than-expected variations in the response of individual communities to MDA implementation, which we refer to as persistent hot spots.^23,31^ This phenomenon is discussed in greater detail in Article 3 of this supplement.^32^ The high degree of variance in response within each study arm meant that although there were, on average, large differences in prevalence and intensity impact (up to 40–60%) between study arms, in our protocol’s primary study outcomes analysis, which was specified before study completion (see Supplement File S2 for details), the between-arm differences in prevalence and intensity effects were not statistically significant. Hence, there was no significant difference between the various schedules of coverage and delivery for MDA. Supplemental Tables S1–S5 in the Supplemental Material files provide the adjusted prevalence ratios and adjusted mean intensity ratios for the arm-to-arm comparisons in each of the SCORE gaining and sustaining studies. The very large intraclass correlations (40%) observed in our studies mean that, if similar studies are performed in the future, it will be necessary to have a much higher number of villages per arm and fewer arms, or a completely different approach, such as a village-by-village evaluation of outcomes.

### Additional analysis of the gaining and sustaining control studies.

Our identification of persistent hot spots as a potential cause of limited overall response to MDA led us to perform an exploratory secondary analysis of community-level features that might explain the location-specific differences in outcomes. Baseline prevalence at the onset of intervention was different from community to community. However, this factor did not predict prevalence or intensity of infection in Year 5 (see Kittur et al.,^32^ in this supplement, and Wiegand et al.,^23^). Reported community-level MDA coverage was also not a good predictor of Year 5 outcomes.^33^ The use of open or unimproved water supplies, close proximity to open water sources, available sanitation (school or home latrines or toilets), and the practice of open defecation did not predict the odds of a community being a persistent hot spot (Musuva RM et al., personal communication). Geospatial clustering was observed for persistent hot spots in western Kenya,^23^ but not in Tanzania or Mozambique. Because the basic village inventory data collected during the SCORE treatment interventions were not revealing, SCORE has now sponsored follow-up “village factors” study in a matched subset of hot spot versus responder communities in Kenya and Tanzania to probe the community characteristics of hot spots in greater detail. Results of these surveys are pending, but we hope to identify the most influential local factors leading to poor response to MDA.

In its 2012–2020 Strategic Plan, the WHO recommended the following schistosomiasis targets: 1) morbidity control by reducing local prevalence of heavy *Schistosoma* infections to < 5% and 2) “elimination as a public health problem” (EPHP) by reducing the prevalence of heavy infections to < 1%. Remarkably, as we took the SCORE MDA implementation to scale, we found that many (52–66%) of the higher prevalence communities already met these criteria for morbidity control or for EPHP at implementation baseline, that is, before any MDA had been delivered. On the other hand, many persistent hot spot communities were unable to achieve the WHO targets for morbidity control despite 4 years of annual MDA with high levels of treatment coverage.

The use of local informants for collection of community-level data on environmental factors related to transmission, including issues such as water and sanitation, proved inconsistent. Similarly, we had concerns about reported treatment coverage data, which were not found to be helpful in explaining the overall results or the emergence of persistent hot spots. Details about these data are found in articles by Binder et al.,^34,35^ in this supplement.

### Impact of MDA on prevalence and intensity of *Schistosoma* infection in first-year students and adults.

Earlier dynamic models had suggested that targeted mass treatment of SAC (who harbor most of the heavy-intensity infections in a typical endemic community) should reduce the force of local *Schistosoma* transmission in proportion to the reductions in overall egg output (i.e., reductions in community-level mean egg counts).^36–38^ Therefore, we looked for evidence of MDA-related changes in local force of transmission by monitoring the levels of infection among younger children (first-year school entrants aged 5–8 years) and among adults aged 20–55 years in communities in gaining control studies, comparing baseline values to those observed in Year 5.

**Figure 5.**
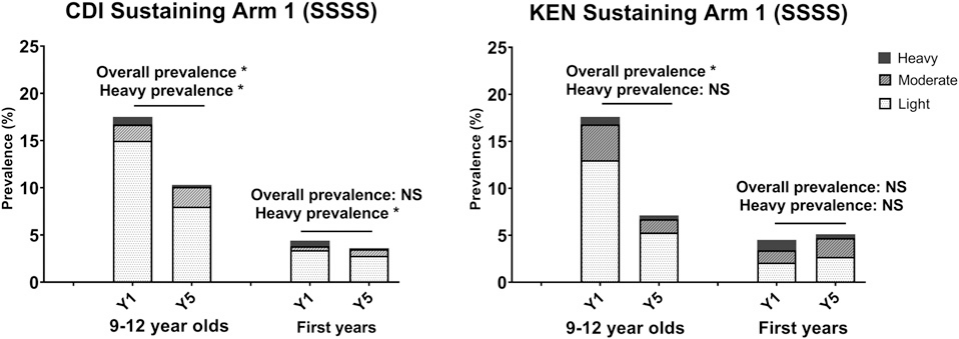
Impact on prevalence of four consecutive years of school-based treatment (SSSS) on 9–12-year-old and first-year schoolchildren in Schistosomiasis Consortium for Operational Research and Evaluation sustaining control studies. Baseline (Y1) and endpoint (Y5) prevalence of *Schistosoma mansoni* infection intensity categories are shown. Asterisks denote statistically significant differences between baseline and endpoint values at a *P* < 0.05 level by χ^2^ testing. CDI = Côte d’Ivoire; KEN = Kenya; NS = not significant.

**Figure 6.**
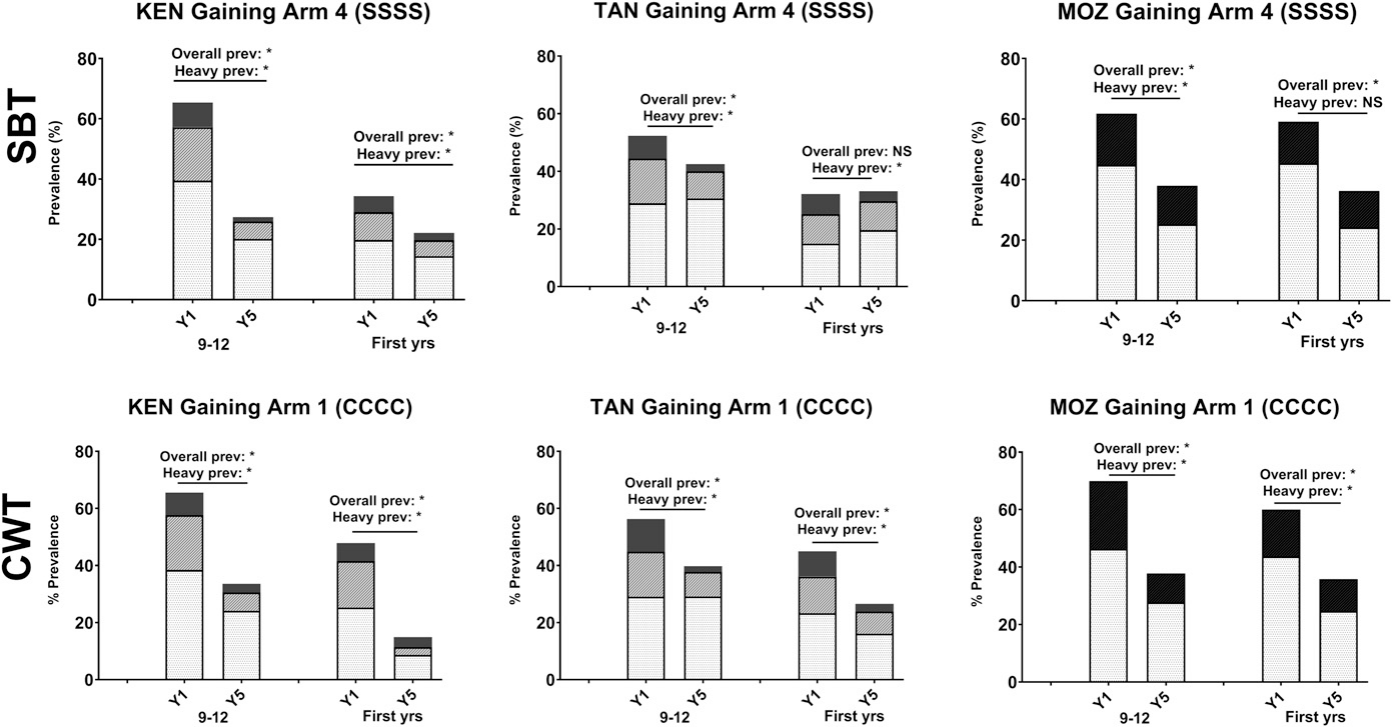
Impact on prevalence of four consecutive years of school-based treatment (SBT or SSSS) or four consecutive years of community-wide treatment (CWT or CCCC) on 9–12-year-old and first-year schoolchildren in Schistosomiasis Consortium for Operational Research and Evaluation gaining control studies. Baseline (Y1) and endpoint (Y5) prevalence of *Schistosoma mansoni* or *Schistosoma haematobium* infection intensity categories are shown. Asterisks denote statistically significant differences between baseline and endpoint values at a *P* < 0.05 level by χ^2^ testing. KEN = Kenya; MOZ = Mozambique; NS = not significant; TAN = Tanzania.

The impact of 4 years of SBT on the nontarget incoming first-year students in sustaining control studies was minimal, with only a statistically significant decrease in heavy infections in Côte d’Ivoire and no impact on those in Kenya (Figure 5). By contrast, in areas of high prevalence that received 4 years of SBT (Figure 6, top row), a more consistent, although not uniform, impact was seen on the incoming first-year students, with a statistically significant decrease in either total prevalence and/or prevalence of heavy infection. In parallel, in areas of high prevalence that received 4 years of CWT (Figure 6, bottom row), the prevalence and prevalence of heavy infections were significantly lower in the nontarget, younger age-group.

**Figure 7.**
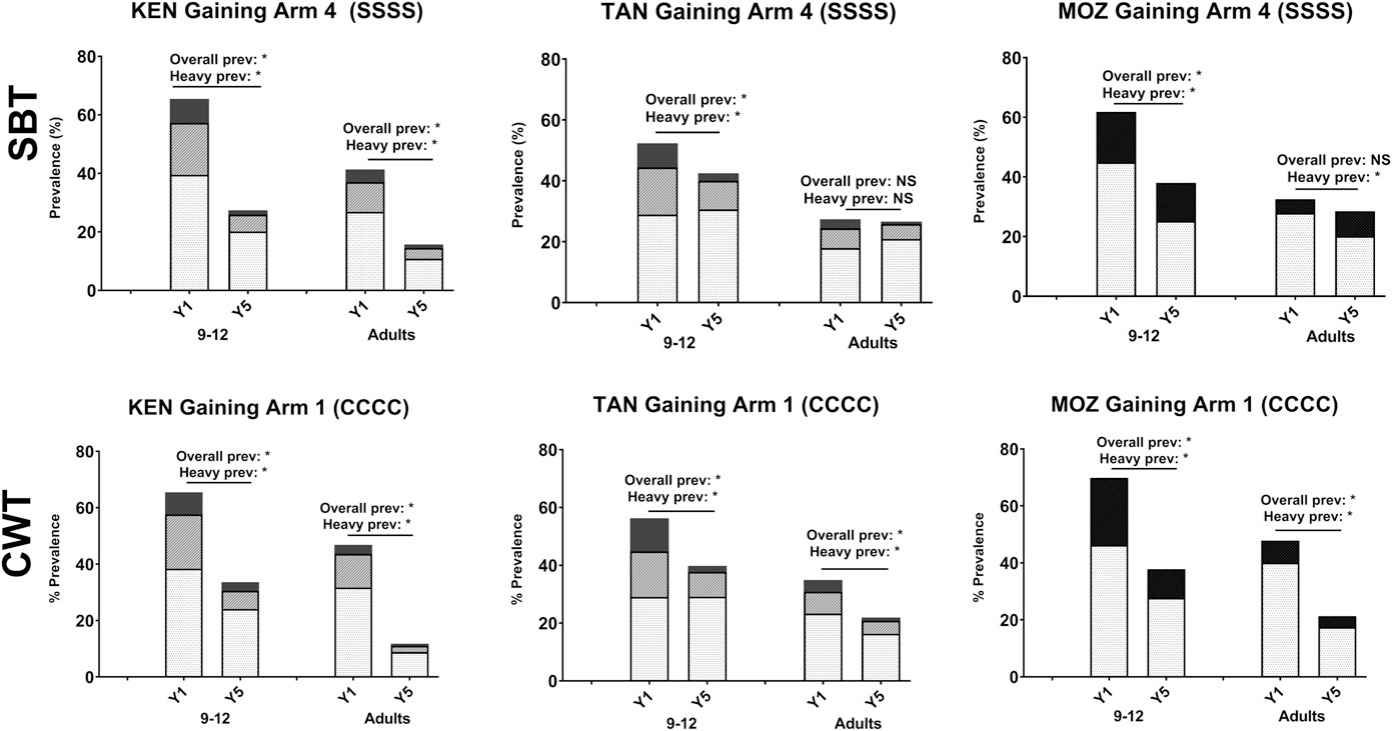
Impact of four consecutive years of school-based treatment (SBT or S; Arm 4) or four consecutive years of community-wide treatment (CWT or C; Arm 1) on 9–12-year-old children and adults in Schistosomiasis Consortium for Operational Research and Evaluation gaining control studies. Baseline (Y1) and endpoint (Y5) prevalence of *Schistosoma mansoni* infection intensity categories are shown. Asterisks denote statistically significant differences between baseline and endpoint values at a *P* < 0.05 level by χ^2^ testing. KEN = Kenya; MOZ = Mozambique; NS = not significant; TAN = Tanzania.

The gaining control studies also evaluated adults aged 20–55 years at the beginning and end of the 5-year study period. In arms that received 4 years of CWT, a modality that included adults in the MDA, both overall prevalence and prevalence of heavy infections were significantly reduced in all studies (Figure 7, bottom row). In arms that received 4 years of SBT only, where MDA did not include adults, the effects on adults varied by study (Figure 7, top row). In the Kenya gaining control study, the overall prevalence and prevalence of heavy infections were significantly reduced among adults in both SBT and CWT arms, indicating that 4 years of SBT was almost as effective as 4 years of CWT in reducing schistosome infection in the local adult population. By contrast, in Tanzania and Mozambique, SBT alone did not reduce schistosome infection in adults.

### Niger study results.

**Figure 8.**
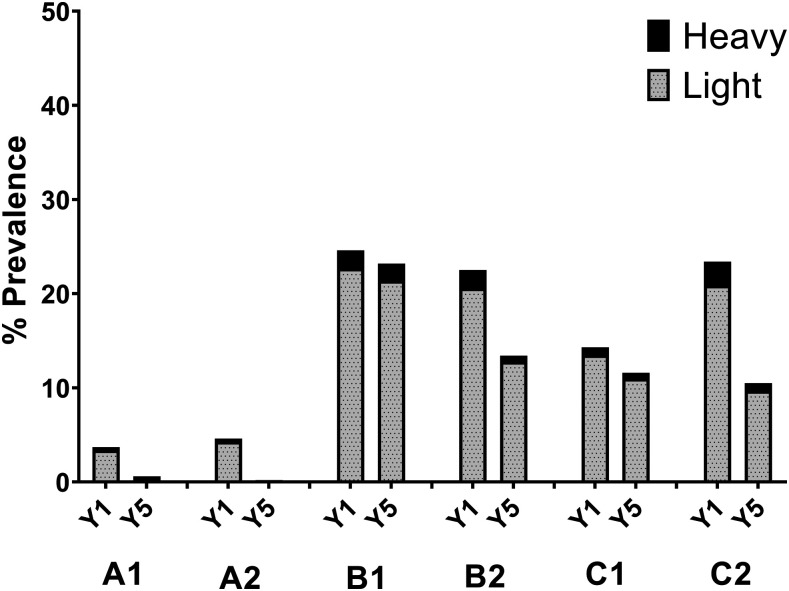
Outcomes of the Schistosomiasis Consortium for Operational Research and Evaluation Niger annual vs. biannual treatment trial. Shown are the starting (Y1) and ending (Y5) prevalences of heavy and light *Schistosoma haematobium* infections in communities that received treatment under the revised Niger study protocol. Studies A (in communities with microhematuria prevalence 5–20% in eligibility testing) and B (communities with microhematuria prevalence > 20% in eligibility testing) began with annual or every-other-year school-based treatment, whereas Study C (communities with prevalence > 20% in eligibility testing) began with annual community-wide treatment during the first 2 years. Each study group was then randomized to receive annual or twice-yearly praziquantel treatments; creating groups A1, A2, B1, B2, C1, and C2 (see Figure 2 for details). The difference in Y5 outcome was significant between groups B1 and B2 (*P* = 0.037). Differences in final prevalence outcomes for studies A and C were not statistically significant.

As shown in Figure 8, biannual SBT treatment was found to be significantly more effective for reducing active schistosome infection than annual SBT in higher prevalence areas (Arm B1 versus B2, defined in Figure 2). However, there was no significant effect of increased treatment frequency in areas with low starting prevalence (Arm A1 versus A2), or in higher prevalence areas that received CWT instead of SBT (Arm C1 versus C2).

## SUMMARY AND PUBLIC HEALTH IMPLICATIONS

To define the effectiveness and test the limitations of current WHO-recommended schistosomiasis control protocols,^9,18^ the SCORE gaining and sustaining operational research projects performed randomized pragmatic trials that compared the impact of different schedules and coverage levels of praziquantel MDA.^1^ This field research was implemented on a much larger scale than any previous trials and under circumstances that would closely mirror implementation of MDA in national control programs. Although there were limitations to the study designs^39^ and performance,^34^ the results from all studies confirm the following:

1. Across all treatment schedules (study arms), MDA with praziquantel significantly reduced local *Schistosoma* infection prevalence and infection intensity among SAC.
2. Evidence from some study locations suggested that outcomes in locations receiving four annual rounds of MDA were better than those in communities that skipped years (i.e., only treated twice in 4 years).^28^ This effect was obscured by the wider-than-expected variation in community-level response to MDA, unmasking the persistent hot spot phenomenon.^23,33^
3. Because individual community responses to standard MDA implementation were so highly variable, with many persistent hot spots, it was likely that some communities did not get the full benefits expected from MDA implementation. Therefore, for optimal MDA results, there is a need to identify persistent hot spots quickly,^30^ well before the 5–6 years currently recommended for treatment impact assessment in the current WHO strategies.^40^ For now, we would recommend at least annual SBT MDA to start, with earlier (Year 3) impact assessments, with ramp up of MDA performance, and nondrug interventions where needed.^30^
4. Although untreated adult populations have been identified as a potential source for persistence of transmission,^41^ the SCORE gaining control studies found that the results obtained by broadening population coverage (i.e., CWT versus SBT) were not better than those obtained with well-implemented SBT. There was evidence that in some moderate-to-high prevalence settings (Kenya, Mozambique, and Niger), SBT may have reduced infection levels among adults and new school entrants through indirect effects on local force of transmission (Phillips AE, et al., personal communication).^24,28,30^
5. None of the MDA schedules evaluated in the gaining and sustaining control studies led to elimination of infection, even in those communities that started at lower prevalence in the sustaining control studies. Therefore, it is likely that future programs aiming for elimination of transmission will need to add extra nondrug interventions (e.g., snail control, improvement in water, sanitation and hygiene, and behavior change interventions) to achieve that next stage of control.

These findings have been discussed in detail with the WHO NTD policy makers, BMGF, and other governmental and nongovernmental NTD control partners to aid in formulation of the next generation of schistosomiasis control and elimination guidelines.

## Supplemental appendix material and tables

Supplemental materials
