# Supplementary material for: Impact of Different Mass Drug Administration Strategies for Gaining and Sustaining Control of Schistosoma mansoni and Schistosoma haematobium Infection in Africa
Source: Am J Trop Med Hyg. 2020 May 12;103(1 Suppl):14–23. doi: 10.4269/ajtmh.19-0829 (PMC7351298; doi:10.4269/ajtmh.19-0829)
Supplement: Supplementary file 1 [file tpmd190829.SD1.pdf]

Supplemental Appendix A: Harmonized Protocol for SCORE Gaining and Sustaining Control Studies

Overall goal: The overall goal of this project is to provide an evidence base for program decisions about mass drug administration (MDA) to gain and sustain control of *Schistosoma haematobium* (Sh) and *Schistosoma mansoni* (Sm) infections. The four studies being funded are:

1. **Study 1 Sh**: Study 1 for *S. haematobium* will compare MDA delivery strategies in study communities/villages with baseline prevalences among school children of 10-24% by filtration or 5-20% by dipstick.
2. **Study 2 Sh**: Study 2 for *S. haematobium* will compare approaches in study areas with prevalences among school children of ≥25% by filtration or ≥21% by dipstick.
3. **Study 1 Sm:** Study 1 for *S. mansoni* will compare MDA delivery strategies in study communities/villages with baseline prevalences among school children of 10-24% by stool examination.
4. **Study 2 Sm**: Study 2 for *S. mansoni* will compare MDA delivery strategies in study communities/villages with baseline prevalences among school children of ≥25% by stool examination.

Study overview: We anticipate projects will be of 5 years duration − 4 years of intervention, with the final data collection occurring in the fifth year. It is likely that MDA will be provided in the fifth year in many communities, but data on the impact of the fifth year of treatment will not be collected as part of SCORE. However, to ensure flexibility to incorporate results of ongoing research and address other changes that may occur during the study period, we will plan to conduct a mid-course evaluation after Year 2, the results of which may affect study plans and funding decisions related to years 4 and 5 of the project. Any possible funding changes will be openly discussed well in advance, and will include significant investigator input.

Research questions and study components

<u>Primary question</u>

What strategy for mass drug administration (MDA) provides the best balance in terms of cost and reductions in prevalence and intensity of *S. haematobium* or *S. mansoni* in school-aged children after 4 years of intervention? Specifically, these studies are designed to answer the questions:

- □ **Sh1:** In communities/villages with *S. haematobium* baseline prevalence 10-24% by filtration or 5-20% by dipstick, what combination of annual school-based treatment (SBT) and drug holidays yields the best outcomes for the lowest cost?
- □ **Sh2:** In communities/villages with *S. haematobium* baseline prevalence >=25% by filtration or >=21% by dipstick,
  - Is there a difference between doing community-wide treatment (CWT) in Years 1 and 2 vs. SBT in those years in terms of parasitologic outcomes after four years?

- After the first two years, what additional benefit accrues from annual MDA vs. providing a drug holiday, and at what cost?
- What is the impact of alternating years of MDA and drug holidays?

□ **Sm1:** In communities/villages with *S. mansoni* baseline prevalence 10-24% by 3 stool examinations with 2 Kato-Katz slides per stool per child, what combination of annual SBT and drug holidays yields the best outcomes for the lowest cost?

□ **Sm2:** In communities/villages with *S. mansoni* baseline prevalence >=25% by 3 stool examinations with 2 Kato-Katz slides per stool per child,
- Is there a difference between doing CWT in Years 1 and 2 vs. SBT in those years in terms of parasitologic outcomes after four years?
- After the first two years, what additional benefit accrues from annual MDA vs. providing a drug holiday, and at what cost?
- What is the impact of alternating years of MDA and drug holidays?

<u>Secondary questions</u>

□ What are the factors that determine the effectiveness of MDA − can we develop reasonable measures of force of transmission that can be used to make decisions about the most cost-effective means of lowering prevalence and transmission in a given village?

<u>Study components</u>

1. *Determine possible study sites.* Use historic and other data to identify communities/villages that are likely to meet criteria for inclusion in the studies. For purposes of this protocol, a study community or village must have a primary school, because several arms of the study are school-based and every participating community must be eligible to be randomized to any of the study arms. However, a study community may have more than one school. If two nearby communities have schools with less than 100 children per school, but they are similar, they can be combined for purposes of this study and be considered as one study community. Two nearby communities that share water sources and/or whose schools have overlapping catchment areas should not be considered two villages for purposes of this study; one of the two should be chosen, although treatment of populations from both villages during the MDA campaign is encouraged, especially if it could create public relations or ethical concerns not to do so.

There is no pre-set population requirement for the size of a community/village, as long as it includes at least 100 schoolchildren between 9 and 12 years of age. In general, preference is for places that have not recently received MDA. If communities/villages have been previously treated, historic treatment data should be included where available. To the extent possible, study communities/villages should be as similar as possible in characteristics that could affect transmission dynamics, including history of past treatment, water sources, etc.

In the case of communities that have more than one school, for determining eligibility for the study (and for follow-up measurements), it is acceptable to choose one school for survey purposes. This school

might be selected based on size, ease of working with the school, or expected rates of infection in school-children. However, MDA should be provided to all schools in the community if it is randomized to the SBT arm.

2. *Gain community support for potential study involvement.* Conduct initial sensitization of communities/villages to gain support for testing school children to determine community eligibility for the study. A simple standard approach will be developed to capture what activities are conducted to achieve sensitization.

3. *Determine eligibility.* Determine eligibility of communities/villages by testing 50 school children in classes that include those ages 13-14. Each Study 1 will require a total of 75 villages (25 per study arm), and each Study 2 will require 150 villages (25 per study arm). Children who test positive in this initial survey should be treated. This will involve examining a mid-day urine in Sh villages, using either dipsticks or 2 filtrations of 10 ml each. In Sm villages this will involve testing a single stool, examining 2 slides per stool.

4. *Randomize.* After an adequate number of eligible communities/villages are identified, they will need to be randomly assigned to the arms of the study − 3 arms for a Study 1 and 6 arms for a Study 2. Regardless of the outcome of randomization, for example, an uneven distribution of a particular factor, villages may not be re-randomized.

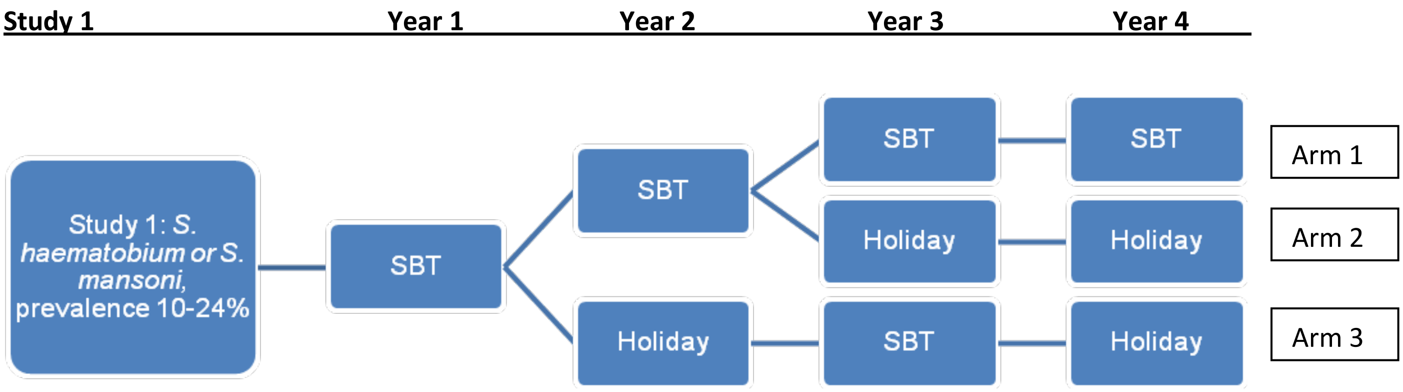

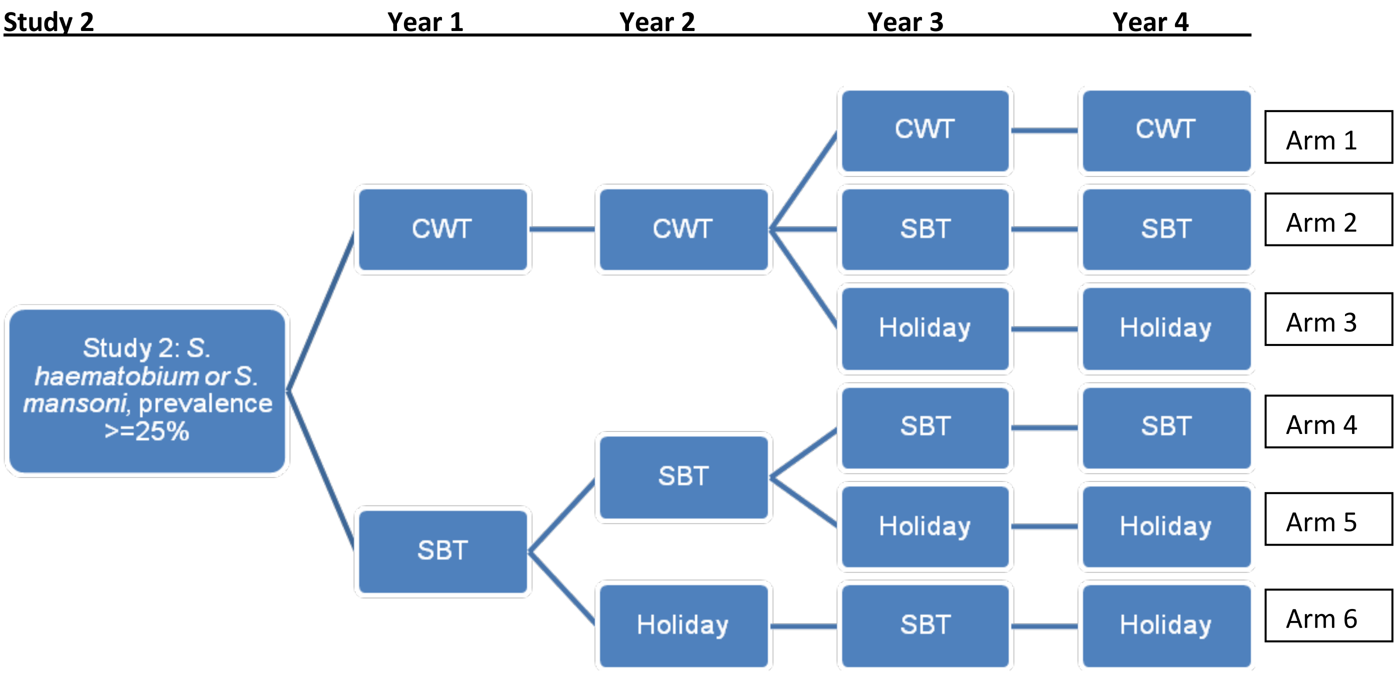

There will be no stratification for randomization of Sh1 and Sm1 communities/villages. Sh2 and Sm2 communities/villages will be randomized with stratification by prevalence on initial screening using Proc Plan, according to an agreed-to protocol.

5. *Identify villages for layered studies (Sh2 and Sm2 only).* Layered studies include subtle morbidity, snail, and parasite genetics evaluations. Investigators funded under this RFP who are conducting Sh2 and Sm2 studies will also be funded to conduct subtle morbidity studies. Investigators funded under this RFP should participate with investigators conducting snail and schistosome population genetics studies to ensure snail collection and plans for parasite collection are in place before MDA.

6. *Sensitize communities.* A greater level of effort will be required to engage eligible communities in the full, multi-year study than was required for the initial eligibility survey. A simple standard approach will be developed to capture what activities are conducted to achieve sensitization.

7. *Train workers.* This includes training on data collection, drug delivery, reporting of adverse events, etc. Incentives may be paid in accordance with local practices and practices of non-government organizations in the area.

8. *Collect baseline and Year 5 data on the study communities/village.* Forms will be provided for use by all studies that will facilitate collection, either by key informants, door-to-door census, or other methods, about important issues that could affect transmission for use at the beginning of the study and in Year 5. These forms will be developed as a collaborative process. They will include such information as water sources, sanitation, occupations, use of antimalarial drugs, etc. It will be more important to have

detailed information (e.g., collected at the individual level, if possible) in villages/communities where subtle morbidity studies are being conducted.

9. *Conduct a census.* A census will be conducted, or quality census data collected via other means, in Year 1 in all selected communities/villages to provide a baseline for estimating coverage. In Sh1 and Sm1 communities, the census may be limited to children 4-18 years of age and their heads of households and parents. In some places, reasonably accurate census data may already be available. For example, in Kenya, community strategy staff may have data already, and in some cases it may be appropriate to use a national census, with or without adjustment. Door-to-door distributors could also potentially conduct the census. If a door-to-door census is being conducted, it is recommended to also get information about latrines and water sources using standard questions (to be provided).

10. *Annually collect information about events that may affect study outcomes*. Because of the complexity of schistosomiasis control, investigators will need to track changes in multiple factors that could affect study outcomes. Randomization is unlikely to address all of these. Factors of concern include but are not limited to changes in sanitation and water supply, unusual weather (e.g. drought and floods) or the effects of climate change, economic development, new dams, political instability or local changes in leadership that affect MDA efforts, changes in the existing health system, community attitudes and behavior related to health and health care seeking, and other programs that are introduced during the study period. A form will be developed for collecting data on an annual basis. This will include suggestions for how to collect information about malaria treatment.

11. *Evaluate schistosomiasis prevalence and intensity at baseline and then annually.* The most important outcomes for the study relate to schistosomiasis prevalence and intensity in the population in all years in which MDA is conducted.

The following populations will be tested:

- In all studies: 100 children in the classes that include those 9-12 years of age. If there are more than 100 children in this age range, select children randomly for testing. For children in this age range in Sm communities, 3 stools will be examined. These prevalence and intensity evaluations will be done each year preceding MDA. (They will not be conducted in communities having a drug holiday.)
- In all studies: 100 first year students in the first and fifth years. For Sm1 and Sm2 studies, it is acceptable to examine 1 stool per child, except in communities doing subtle morbidity studies, where 3 stools per child will be collected. If samples for 100 first year students cannot be obtained, test as many as possible. Do not add age groups.
- In Sh2 and Sm2 studies: 50 adults ages 20-55 years from the community in the first and fifth years. For Sm2 studies, it is acceptable to examine 1 stool per adult, except in communities doing subtle morbidity studies, where 3 stools per adult will be collected. A variety of processes to identify these adults will be acceptable. A subgroup will work to articulate these. They could range from a random sample of adults identified through the census to a random selection of areas within the study village and random selection of adults from within those, to other options. Only one adult per

household should be selected, and pregnant women are eligible. SCORE does not require adults to be tested in Sh1 and Sm1 studies.

In Sh1 and Sh2 studies, a single mid-day urine should be tested, with eggs quantified and recorded separately for two 10-ml filtrations. In Sm1 and Sm2 studies, 2 slides per stool should be examined using Kato-Katz. It is acceptable to collect stools for Sm1 and Sm2 studies but defer reading them until later.

12. *Conduct MDA and record serious adverse experiences (SAE).* Praziquantel (PZQ) will be provided as a single dose of 40 mg/kg, using a dose pole, using the approach defined by the study arm. Normal PZQ treatment exclusion criteria apply. MDA will not be provided during drug holidays.

School-based treatment (SBT)

During school-based treatment, it is expected that PZQ will be administered by trained teachers to all primary school-age children. Children in all schools in the community should be treated, even if they are not in a school where children are being tested. Any time school-based treatment is occurring, practical efforts should be made to treat non-school attendees who span the same age range as children who are in school e.g. community sensitization and mobilization efforts, radio announcements, and other Information, Education, and Communication strategies. However, major treatment strategies outside the school-based venue should not be implemented. Investigators should document school attendance rates throughout the study community.

Community-wide treatment (CWT)

CWT for these SCORE studies means providing treatment to the entire eligible population, which only excludes children under 4 years of age or under 94 cm in height, in the study community. A checklist will be provided that can be used to describe the ways in which CWT is provided. If coverage after the first attempt is less than 75%, additional efforts should be made to increase the coverage. It is essential that treatment be directly observed.

Regardless of method of distribution, those providing PZQ will be required to keep records consisting of names, ID numbers, age, sex and height (for children), numbers of tablets, etc. Systems must be in place for responding to and reporting SAEs. In villages receiving SBT, SAEs should be reported to teachers. In villages receiving CWT, SAEs should be reported to community distributors. Teachers and community distributors, respectively, should be trained on whom to contact should there be a serious SAE. Reporting will be in accordance with WHO procedures. Minor complaints and side effects that are not serious will not be measured.

Sample forms will be provided for recording information about who receives treatment. These will include name, age, weight, height, etc.

It is recognized that some populations are migratory. There will not be special attempts to follow up or otherwise reach migrant populations.

Excess PZQ will not be ordered for purposes of leaving in clinics. However, partially used tins will be left with teachers for use during the year as needed or for children not in attendance the day of treatment.

13. *Estimate coverage and achieve targets.* Estimations of coverage should use an appropriate denominator, derived from census data. The goal for MDA in this study is 100% coverage. In agreeing to do this study, researchers are committing to achieve at least 90% coverage of children enrolled in school and at least 75% coverage of school-aged children overall. For CWT, the investigators are committing to achieve at least 75% coverage of the entire community. This may require repeated efforts if it is not achieved through the first round of intervention. If coverage is less than 100%, the reasons for this should be determined and recorded.

14. *In Year 2, estimate costs.* In Year 2, investigators will be expected to collect data using a standard protocol that will be established with investigator input. Sites that have already been collecting data as a part of the Gates-funded effort to understand the costs of integrated programs for neglected tropical diseases will be able to use much of the data they have already collected as the basis for the cost component of this study. However, some additional variables may need to be collected, and this will be discussed in the first annual meeting, during the development of the standard data collection protocol with investigators.

15. *Share samples and data.* All SCORE investigators will be expected to collaborate and to facilitate the collection, analysis and sharing of samples and data. Investigators are not expected to include these parallel studies in their budgets, but should be aware of them and may need to provide budget estimates to applicants for these other funds and to SCORE, for example, to collect additional samples.

16. *Disseminate results.* Funded investigators will be encouraged to publish their findings as appropriate to ensure public access. They will be expected to agree to provide their data analysis and publication plans to and to work cooperatively with the SCORE secretariat and other SCORE-funded investigators. Draft manuscripts should be submitted to the SCORE secretariat at least 2 weeks prior to submission to a journal for publication in order to ensure optimal coordination.

Supplemental Material
Statistical Analysis Plan (SAP) for
SCORE Studies of Gaining and Sustaining Control of Schistosomiasis, and Cohort Studies of *Schistosoma* Infection-Associated Morbidity

Version 11.5, 25 Jul 2017

Authors: Charles King, Michelle Clements, Ye Shen, Sue Binder, and the SAP Working Group[1]

PREFACE

This statistical analysis plan (SAP) has been developed by SCORE team members to harmonize the evaluation of outcomes data from SCORE's 'Gaining and Sustaining' schistosomiasis control studies in Cote d'Ivoire, Kenya, Mozambique, and Tanzania, and the Cohort studies of *Schistosoma*-associated morbidity in Kenya and Tanzania. It provides guidance on appropriate data sources, variable definitions, and approaches for assessing quantitative outcomes. The intent is to provide comparable analysis of country-level data for each of these SCORE studies. The data analysis plan presented below and in the Appendices is based on statistical methods that are available using SAS. Use of other statistical analysis packages is also appropriate.

This SAP covers the studies as registered, i.e., it does not cover combined studies; however, it is strongly recommended that combined analysis be conducted for the two Sm1 studies, the two Sm2 studies, and the *S. mansoni* arms that involve SBT (Arms 1-3 from Sm1 and Arms 4-6 from Sm2).

The contents of this SAP represent a minimum analysis for each study. The tables described herein should be completed and provided to SCORE before the conclusion of the study for SCORE to use in reporting to the Bill & Melinda Gates Foundation. They should be available for sharing upon appropriate request, e.g., as supplemental tables for publications. The choice of data to present in published papers is left to authors and will reflect country-specific needs and interests; however, the definitions and approaches described in this document should be used in analysis and published results should be consistent with those from the SAP analyses. In addition, investigators should review the guidelines from CONSORT (Campbell 2012) to ensure that they address all requirements for reporting randomized trials. Additional exploratory analyses beyond those described here are encouraged.

Table of Contents

PREFACE 1

I. STUDY ABBREVIATIONS AND BRIEF DEFINITIONS 3

II. GOALS AND OBJECTIVES OF THE SCORE GAINING AND SUSTAINING AND COHORT STUDIES 4

a. Overview of SCORE Gaining and Sustaining studies 4

[1] Carl Campbell, Jennifer Castleman, Dan Colley, Jan Hattendorf, Nupur Kittur, Sue Montgomery, Annette Olsen, Steve Rathbun, Christopher Whalen, Ryan E. Wiegand

b. Overview of SCORE Cohort studies of infection-associated morbidities....6
c. Study design: Gaining and Sustaining studies....6
d. Study design: Cohort studies of *Schistosoma*-associated morbidity....7
III. DATA COLLECTION, AND TYPES OF DATA AVAILABLE....8
a. Individual-level data in Gaining and Sustaining studies....8
b. Individual-level data in Cohort studies....9
c. Village-level data....9
IV. SCORE UNIFORM DATASETS....10
V. DEFINITIONS OF PREVALENCE, INTENSITY, AND COVERAGE....11
VI. STUDY QUESTIONS AND END-POINTS OF INTEREST FOR GAINING AND SUSTAINING STUDIES....12
a. Key research questions for Gaining and Sustaining studies....12
b. Analyses, tables, and figures to be reported to SCORE by Gaining and Sustaining study researchers13
Table 1. Baseline characteristics of participants, by arm. 13
Figure 1. Map of study villages, by arm. 13
Table 2. Coverage and harms. 13
Figure 2. Study flow by arm. 13
Table 3. Descriptive results for baseline and Year 5. 13
Figure 3. Intensity categories, by Arm, by Year. 14
Table 4. Comparisons of prevalence and intensity at Year 5 among arms....14
Figure 4. Village-level mean intensity by year, by arm. 17
VII. STUDY QUESTIONS AND END-POINTS OF INTEREST FOR COHORT STUDIES....17
a. Key research questions....17
b. Analyses, tables, and figures to be reported to SCORE from Cohort study researchers....18
Table 1. Baseline and Year 5 characteristics of participants, and loss to follow-up, by arm....18
Table 2. Year 1/Year 5 Comparison of Prevalence and Intensity, by Arm....19
Figure 1. Study flow by arm. (See instructions for Figure 2 in the description of tables and figures for cross-sectional analysis.)....19
Table 3. Descriptive results on morbidity markers, by arm, by year. 19
Tables 4 (multiple tables). Comparison between Arm 1 and Arm 6 of Year 5 morbidity outcomes....20
Tables 5 (multiple tables). Comparison between Arm 1 and Arm 6 of change in morbidity outcomes from Year 1-Year 5. 21
c. Missing cohort data....22

Table 6. Comparison of imputed and unimputed values for outcomes with >10% missing data in Year 5 (from unadjusted models). 23

d. Additional SAS code 23

VIII. PROTOCOL DEVIATIONS AND OTHER ISSUES 23

REFERENCES 24

APPENDICES 25

A. Harmonized Protocol for SCORE Gaining and Sustaining Control Studies 25

B. Harmonized Protocol for SCORE Cohort Studies of Subtle Morbidity 25

C. Definitions for SCORE Studies 25

D. Data Elements Available for Analysis 25

E. Data Dictionary for SCORE Gaining and Sustaining Control Studies 25

F. Data Dictionary for SCORE Cohort Studies 25

G. Data Dictionary for Coverage 25

H. Initial Analysis Plan for SCORE Gaining and Sustaining Control Studies 25

I. Example of a Study Flow Diagram 25

J. Initial Analysis Plan for SCORE Cohort Studies of Subtle Morbidities 25

K. Additional SAS Code for Analysis of Cohort Data 25

L. Protocol Deviations 25

I. STUDY ABBREVIATIONS AND BRIEF DEFINITIONS

CWT – Community-wide treatment

Hb – Hemoglobin

Epg – eggs per gram

GEE - Generalized estimating equations

ICC – intra-class correlation

MDA – Mass drug administration delivered on an annual basis at a target dose of 40 mg/kg, with dose estimated from subject height using a dosing pole

SAC – School age children

SBT – School-based treatment

Sh – *Schistosoma haematobium*

Sh1 – Sh studies of Sustaining control, in communities found to have prevalence <25% among 13-14 year-old children during eligibility screening

Sh2 – Sh studies of Gaining control, conducted in communities found to have prevalence ≥25% among 13-14 year-old children during eligibility screening

SCORE — Schistosomiasis Consortium for Operational Research and Evaluation

Sm – *Schistosoma mansoni*

Sm1 – Sm studies of Sustaining control, conducted in communities found to have prevalence <25% among 13-14 year-old children during eligibility screening

Sm2 – Sm studies of Gaining control, in communities found to have prevalence $\geq$25% among 13-14 year-old children during eligibility screening

SUDS – SCORE Uniform Data Set

Y – Year: Y1 data collection is defined as the baseline data collection, which occurs before treatment. Y1 treatment refers to the first MDA treatment

<u>Abbreviations related to study arm treatment sequences</u>

c – CWT

h- holiday

s – SBT

ssss – treatment sequence for Sh1/Sm1 Arm 1 and Sh2/Sm2 Arm 4

sshh – treatment sequence for Sh1/Sm1 Arm 2 and Sh2/Sm2 Arm 5

shsh – treatment sequence for Sh1/Sm1 Arm 3 and Sh2/Sm2 Arm 6

cccc – treatment sequence for Sh2/Sm2 Arm 1

ccss – treatment sequence for Sh2/Sm2 Arm 2

cchh – treatment sequence for Sh2/Sm2 Arm 3

II. GOALS AND OBJECTIVES OF THE SCORE GAINING AND SUSTAINING AND COHORT STUDIES

The overall goal of the SCORE project is to provide an evidence-base and tools for programmatic decisions on how best to gain and sustain control of *Schistosoma haematobium* (Sh) and *Schistosoma mansoni* (Sm) infections, and, ultimately, to eliminate them. The protocols for the SCORE Gaining and Sustaining Control and Cohort studies – which are randomized trials designed to inform approaches to mass drug administration (MDA) – were developed through a collaborative process. The harmonized protocols, which were to be followed by all groups funded to conduct this work, appear in Appendices A (Gaining and Sustaining studies) and B (Cohort studies). Appendix C describes the original study term definitions. Appendix D is a table describing the data elements available for analysis from these studies, by country and study.

a. Overview of SCORE Gaining and Sustaining studies

The SCORE Gaining and Sustaining study protocol described the four types of studies to be conducted:

1. **Study Sm1:** Study 1 for *S. mansoni* was a cluster-randomized trial that compared MDA delivery strategies in study communities/villages with prevalence during eligibility testing of 10-24% by Kato-Katz stool examination.
2. **Study Sm2**: Study 2 for *S. mansoni* was a cluster-randomized trial that compared MDA delivery strategies in study communities/villages with prevalence during eligibility testing of $\geq$25% by stool examination.

3. **Study Sh1**: Study 1 for *S. haematobium* was a cluster-randomized trial that compared MDA delivery strategies in study communities/villages with prevalence during eligibility testing of 10-24% by urine filtration (or 5-20% by urine dipstick test for hematuria).
4. **Study Sh2**: Study 2 for *S. haematobium* was a cluster-randomized trial that compared approaches in study areas with prevalence during eligibility testing of ≥25% by filtration or ≥21% by dipstick.

For purposes of this protocol, a study community or village was required to have a primary school, because several arms of the study are school-based and every participating community must be eligible to be randomized to any of the study arms. However, a study community could have more than one school. If two nearby communities had schools with fewer than 100 children per school, but they were similar, they could be combined for purposes of this study and be considered as one study community. Two nearby communities that shared water sources and/or whose schools had overlapping catchment areas were not to be considered two villages for purposes of this study; one of the two could be chosen.

Prior to enrollment, village eligibility was determined by testing fifty 13-14 year-old children in each village. Once a village was enrolled in the study, the **primary outcomes of interest are prevalence and intensity among 9-12 year-old children**. A simple randomization procedure was used, without stratification. Re-randomization based on the first randomization results was not permitted.

In addition to 9-12 year-old children, SCORE studies included some data collection on first-year students and, in Sh2 and Sm2 studies, in adults. More information about data collected from these populations is provided in the protocols and in Appendix D. Their results are not considered in this SAP, but may be used for future secondary analyses of the SCORE gaining and sustaining studies.

Data are available for the following studies:

- Sm1: Cote d'Ivoire, Kenya
- Sm2: Kenya, Tanzania
- Sh2: Mozambique

Limitations to the analyses included in this SAP:

- Sh1 and Sh2 studies to be conducted in Niger had to be redesigned because of a failure to randomize appropriately and will not be considered further in this SAP. Thus, there are no Sh1 studies covered in this SAP.
- Adverse events during MDAs were recorded by those administering the drugs in a manner consistent with WHO or country guidelines. These records were not requested as part of the SCORE research. Therefore, the planned SAP analyses do not include a "safety" component.
- Requested cost data were not recorded in a systematic manner across countries. Therefore, the SAP analyses do not include any cost-effectiveness components.

b. Overview of SCORE Cohort studies of infection-associated morbidities

In addition to the Gaining and Sustaining studies, Cohort studies were started in a minimum of 8 randomly selected villages participating within each of the SCORE Sh2 and Sm2 projects, in order to determine whether the intensity of village-level MDA intervention affects measures of health and well-being among school age children. The overall goal of these studies is to provide information to policy- and decision-makers about the actual impact on morbidity of alternative strategies to multi-year MDA with praziquantel in schistosomiasis-endemic areas. Because Niger did not randomize according to the study protocol, and because of poor participation and large loss to follow-up in Mozambique, only data from Kenya and Tanzania Sm2 Cohort studies are available for analysis. Thus, no longitudinal cohort study data are available for Sh, and Sh morbidity will not be considered further in this SAP.

c. Study design: Gaining and Sustaining studies

Overview: The Gaining and Sustaining studies are parallel cluster-randomized, open-label operational research trials of praziquantel MDA for control of prevalence and intensity of *Schistosoma* infections in endemic communities in Africa.

Study Arms: Study 1 has three study arms arranged as follows:

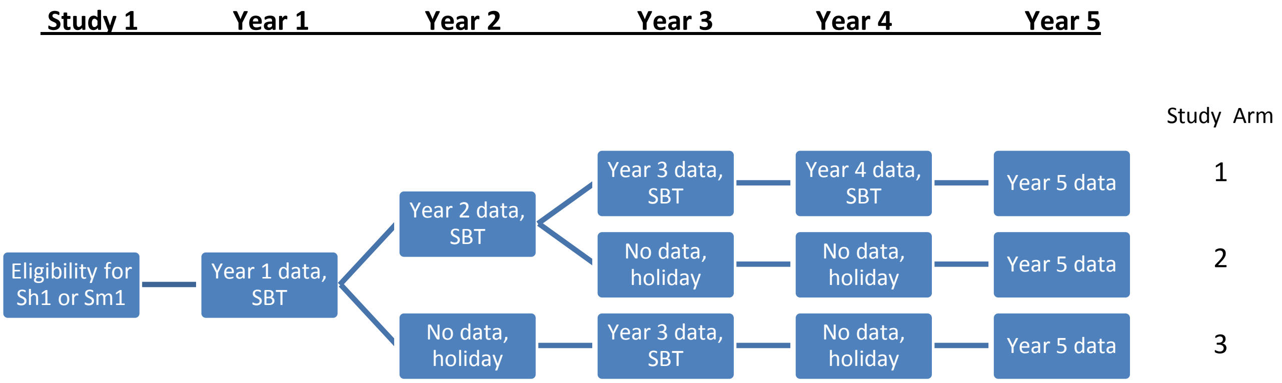

Study 2 for both species has six study arms arranged as follows:

Study 2 Year 1 Year 2 Year 3 Year 4 Year 5

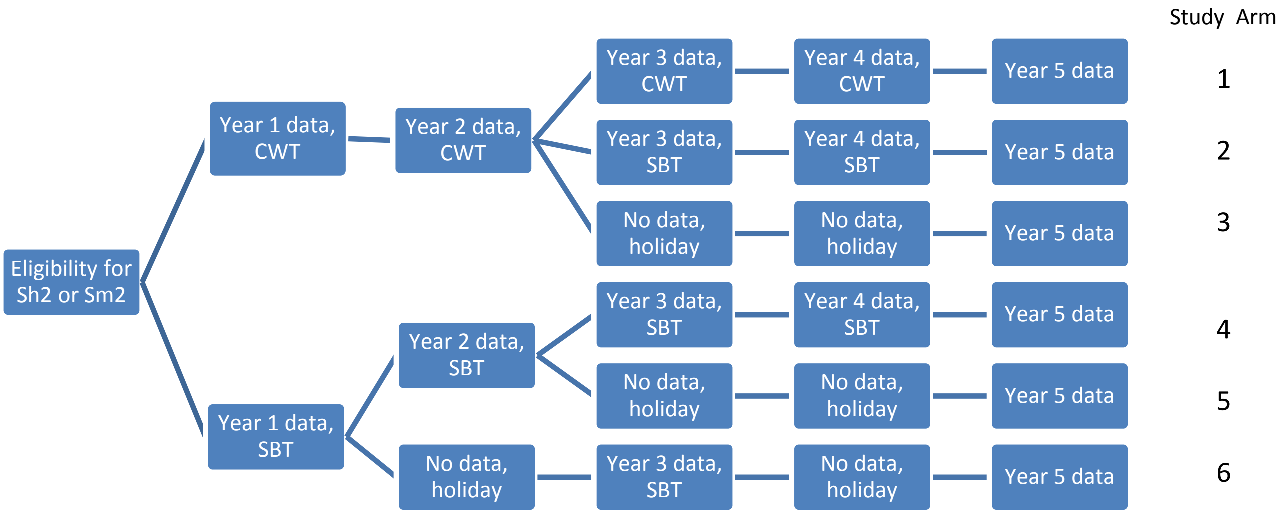

Note that Arms 1, 2, and 3 in Study 1 correspond to Arms 4, 5, and 6 in Study 2. Holidays indicate years in which a village did not receive MDA with praziquantel. No testing of children was conducted during holiday years; otherwise, infected children would have required treatment, which would have precluded evaluation of the impact of MDA holidays.

Because six villages in Mozambique had their cross-sectional study arm re-assigned after randomization (see Appendix L for Protocol Deviations), the initial analysis of treatment outcomes will be "as treated." Children included in analysis are those who are an "available case," that is, someone who provided age and sex information and at least one slide for evaluation of infection status.

Populations and expected sample size: The goal of each country study was to enroll 25 eligible villages per study arm, and to monitor each village's prevalence and intensity of *Schistosoma* infection (whether Sm or Sh) among **a random sample of 100 resident 9-12 year-old children each year**, starting before implementation of MDA (Year 1) and continuing through Year 5 (see study arm schema above). Nominal enrollment for each Study 1 was 75 villages, with 7,500 children tested each year. Nominal enrollment for Study 2 was 150 villages, with 15,000 children tested each year.

d. Study design: Cohort studies of *Schistosoma*-associated morbidity

Overview: The Cohort studies are sub-studies within SCORE Gaining Control studies, i.e., in communities with $\geq$25% prevalence of schistosomiasis during eligibility testing. They involve only Arms 1 and 6 of the Gaining Control studies, selected to represent the most intensive and a much less intensive intervention sequence, respectively (see below). Enrolled children were followed for five years, with evaluation occurring at Years 1 (baseline), 3 (after two MDA treatments), and 5 (after four MDA treatments).

Cohort study design and expected sample size: Cohort studies aimed to enroll 100 children from at least 4 communities/villages in each study arm, for a total of 800 children per study. The two Sh2 cohorts were discontinued: Mozambique because of significant loss to follow-up and Niger because of inappropriate randomization.

Cohort Study Year 1 Year 2 Year 3 Year 4

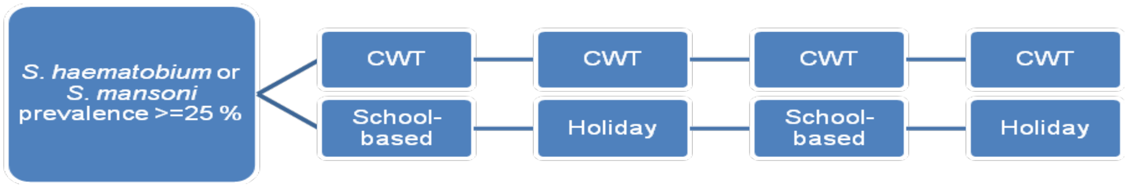

Villages for inclusion in this study were meant to be a random sample of all the villages in the two study arms of interest (Arms 1 and 6); however, to facilitate the survey work investigators were allowed to select them randomly from among a restricted group of no less than 10 out of the 25 villages in each arm, chosen, for example, to exclude very remote villages.

The protocol called for selecting the 100-child cohort from the school class that starts at 7 or 8 years of age. If there were over 100 children in this class, children were to be randomly selected for participation. If this class did not include 100 children, then all of the children in that class should have been enrolled along with a random selection of children in the next older class, in order to reach the total of 100 study subjects.

III. DATA COLLECTION, AND TYPES OF DATA AVAILABLE

The table in Appendix D describes the data elements available from the SCORE Gaining and Sustaining and Cohort studies.

a. Individual-level data in Gaining and Sustaining studies

Parasitological data on *Schistosoma* infection among children ages 9-12 were collected prior to each MDA and a year following the last treatment. No data were collected in villages during a holiday year.

In the Sm studies, duplicate Kato-Katz slides were made from each of three daily stool samples from each individual, and the number of eggs found in each slide was recorded. In the Sh studies, 10 ml of urine from a single well-stirred urine sample for each individual was filtered twice, and each of these two filtrations was examined under the microscope; the number of eggs found in each slide and the corresponding volume of urine filtered was recorded.

Parasitological data were also collected in Years 1, 3, and 5 on children in the first-year class and in Years 1 and 5 on adults (see Appendix D). However, given that data on these populations do not represent the

primary end-points of the SCORE treatment studies, and that the data collected on them are much more limited than that for 9-12 year olds, they will not be reported as primary end-points.

b. Individual-level data in Cohort studies

Primary Morbidity Markers: The following were measured in Years 1, 3, and 5, except for ultrasound, which was to be conducted in Years 1 and 5. Kenya chose to perform abdominal ultrasounds in Year 3 as well. All the markers will be available in the SCORE uniform datasets (SUDS) as described in section IV. For details on variable coding, please refer to Appendix F of this SAP: 'Data Dictionary for SCORE Cohort Studies.'

1. Height in cm and weight in kg transformed to age-adjusted Z-scores for each outcome using established WHO/CDC standards, and presence or absence of stunting or wasting.
2. Blood hemoglobin (Hb). While collection of a venous sample was preferred, finger prick capillary blood was acceptable Based on WHO criteria, at sea level and up to 1000m elevation, anemia is defined as Hb < 11.5 g/dL for age < 12 years, Hb < 12 for females > 12 years, Hb < 12 for males 12-15, and Hb < 13 for males > 15. Altitude-related changes in normal circulating hemoglobin levels require adjustment of anemia cutoffs for higher elevations. The cutoffs are 0.2 gm/dL higher for each age group in Tanzania and Kenya locations as they are located at 1000-1250m elevation [1].
3. Physical fitness (VO2) based on the 20-meter shuttle run protocol. VO2 is calculated as follows:
   (1) Speed= 8 + .5 x No. shuttles successfully completed;
   (2) VO2= 31.025 + 3.238 * Speed – 3.248 * Age in years + .1536 * Speed * Age in years.
4. Measures of well-being based on the standardized questionnaire, PedsQL. This instrument was translated and validated prior to local use. The Physical and other subscale scores for PedsQL were based on child's graded responses to each question. Scale scores were calculated by 1) reversing item answer scores and linearly transforming to a 0-100 scale [0=100, 1=75, 2=50, 3=25, 4-0], and 2) for each subscale, adding transformed scores and dividing by number of items scored. If over 50% of items in the subscale are missing, the subscale is coded as missing. C in the variable name indicates child responses, P indicates parent.
5. Abdominal ultrasound in Years 1 and 5. (Detailed information on variable coding can be found in Appendix F.)

Infection status (Secondary markers): All children in the cohort were to be tested in Years 1, 3, and 5 for *S. mansoni* infection. For all children in Sm2, 3 stools were examined using Kato-Katz, 2 slides per stool. For accurate calculation of age-in-months used in anthropometric scoring, exact date of birth was to be ascertained for all participating cohort children.

c. Village-level data

Village-level data were collected about usual water sources, sanitation, and other issues by key-informant interviews. Coverage data were collected following each MDA. In general, the data on numbers receiving treatment was provided by those administering treatment – either school personnel or community health workers. In some projects, such as in Kenya Sm1 and Sm2, MDA was carried out by the study team. In other places, such as in Cote d'Ivoire, the national program administered the MDA. Denominator data came from

a variety of sources, including governmental census reports, village leader estimates, and door-to-door surveys.

In SBT villages, coverage was recorded for SAC. In CWT villages, coverage values for both SAC and the entire population were requested.

In all studies except Cote d'Ivoire, Year 2 cost data were collected in a sample of at least 5 representative SBT villages in each study. In Cote d'Ivoire, data were collected in Year 3. In Sh2 and Sm2 studies, data were collected in at least 5 representative CWT villages as well. Data included information on personnel; transport; and consumables, materials, and services. Investigators were asked to capture program costs and not the costs of research.

Limited data are also available on snail quantities, species, and schistosomes population genetics from eight villages from Arm 1 and eight villages in Arm 6 in Tanzania.

Because of variability in data capture, secondary analyses of costs and the influence of village-level and parasite strain factors are not included as part of this SAP. These analyses will be developed separately by the individual investigators.

IV. SCORE UNIFORM DATASETS

SCORE used standardized approaches to data cleaning and creation of analytic data sets. Data were cleaned in country based on a series of checks provided by SCORE. "Clean" data were sent to SCORE for further assessment, and issues identified with the data (e.g., missing data, data that appeared to be out of range, etc.) were sent back to the country to be corrected to the extent possible. One value that appeared clearly wrong – a weight of 12 kg for a 9-year-old child – was re-coded as missing.

Rules employed in the SUDS datasets include:

- Individuals who did not have parasitological data recorded were entirely excluded from the datasets.
- Inclusion in the age groups: 5-8 years, 9-12 years, and adult was based on recoded ages, except for adults in some Kenya data who were documented to meet the 20-55 age requirement but did not have exact ages recorded. Mozambique enrolled many children with recorded ages outside the study requirements in the belief that these children were likely of appropriate ages for inclusion in the study. If the recorded age did not meet study criteria, these were classified as "Other."

Once clean, data were placed in the SCORE Uniform Data Set (SUDS) format, and returned to the country. Datasets for each study are stored separately. (Each country has only one study, except for Kenya, whose two studies (Sm1 and Sm2) are stored in individual datasets).

Each data set is available in SAS, Excel, and *.csv formats, and is accompanied by the appropriate SUDS data dictionary. The data dictionary also includes metadata pages specific for each study site, indicating issues identified during data collection and cleaning that may need to be taken into account in analysis.

SUDS data dictionaries for Sh and Sm Gaining and Sustaining studies and Cohort studies are in Appendices E and F, respectively. The SUDS data dictionary for coverage is in Appendix G.

V. DEFINITIONS OF PREVALENCE, INTENSITY, AND COVERAGE

The following definitions will be used for individual-level data:

- Individual mean eggs per gram (epg) (Sm studies): 24 * total number of eggs found in all slides / number of slides examined. Individual mean epg will be used for reporting, as it is the standardly used metric for infection intensity.
- Individual egg count (Sm studies): Total number of eggs found in all slides examined / number of slides examined. Individual egg count will be used for analysis, as epg does not have a continuous distribution. Note that some software packages may require individual egg count to be rounded prior to analysis. If the analysis requires integers, then the egg count used for formal statistical analysis should be 6 * Total number of eggs found in all slides examined / number of slides examined, rounded to the nearest whole number. If estimated counts are over 1,000 after adjustment for number of slides, they should be truncated at 1,000, as is common practice in schistosomiasis clinical research studies.
- Individual mean eggs per 10 ml (Sh studies): Number of eggs found * 10 / volume of urine filtered. If estimated counts are over 1,000 after volume adjustment, they should be truncated at 1,000. Mean eggs per 10 ml will be used for both analysis and reporting, as this measure is both continuous and a standard metric. Note that some software packages may require mean eggs per 10 ml to be rounded prior to analysis, but this will only affect individuals with less than 10ml of urine filtered.
- Egg positive: a child will be deemed to be egg-positive if one or more eggs were found in any of the slides examined.

The following definitions will be used for reporting on cross-sectional and cohort studies:

- **Prevalence of *Schistosoma* infection:** Percentage of egg-positive children among the 9-12 year olds tested in each community each year.
- **Mean intensity of *Schistosoma* infection:** *Arithmetic* mean of individual mean epg or eggs per 10 ml among the 9-12 year-old children. Two values are to be reported:
    - i) Village-level intensity: This is the mean egg count for all tested 9-12 year-old subjects (including those with zero egg counts), which is a measure of community-level contamination potential.
    - ii) Individual-level intensity: This is the mean egg count among egg-positive subjects, which is an estimate of the intensity of infection among known active cases.
- **SBT coverage:** Numbers of school-age children treated / numbers of school-age children.
- **CWT coverage**: Numbers of people in the community treated / number of treatment-eligible people in the community.

VI. STUDY QUESTIONS AND END-POINTS OF INTEREST FOR GAINING AND SUSTAINING STUDIES

The following study questions and endpoints of interest are derived directly from the objectives as stated in the original, harmonized SCORE protocol.

a. Key research questions for Gaining and Sustaining studies

The primary research questions are:

1a. Does the final (Year 5) prevalence of schistosomiasis among children age 9-12 differ by Study Arm?

1b. Does the final (Year 5) mean intensity of Sm or Sh infection among all children aged 9-12 differ by study arm?

More specifically, the planned analysis will involve a series of arm-to-arm comparisons, prioritized according to what questions are likely to be most important for decision-makers. Because there is risk of “false discovery” of statistically significant association when multiple comparisons are made (Type I error), the number of comparisons formally tested will be restricted. In both studies, the focus of analysis will be on comparison of the current standard of care with alternative treatment strategies in decreasing order of priority. P value cutoffs will not be adjusted. However, this potential limitation of the analyses will be included in the discussion of results, and interpretation will be guided by the strength of the arm-specific effects.

In Sm1 and Sh1 areas, the standard for morbidity control in relatively low-prevalence areas is annual SBT (Arm 1). Instituting “holidays” is hypothesized to lessen the impact of a treatment program on prevalence and intensity. This yields the following comparisons in priority order:

1. Because alternate years of SBT and Holidays would be the easiest to institute, the first comparison in Sm1 should be between **Arm 1 and Arm 3**.
2. Subsequently, we will compare **Arm 1 and Arm 2.**

For high prevalence communities, the standard for schistosomiasis control is annual SBT, as conducted in Arm 4 of the Gaining and Sustaining studies. Instituting CWT may increase the impact of treatment on local prevalence and intensity of *Schistosoma* infections. Under this consideration, the following comparisons are to be considered for the Sm2 and Sh2 trials:

1. Because annual SBT is the standard, the first comparison should be between **Arm 1 and Arm 4**.
2. Subsequently, we will compare **Arm 1** and **Arm 2**, **Arm 1** and **Arm 3**, **Arm 4** and **Arm 5**, and **Arm 4** and **Arm 6**.

The original statistical analysis plan for evaluating the Gaining and Sustaining studies is found in Appendix H.

b. Analyses, tables, and figures to be reported to SCORE by Gaining and Sustaining study researchers

The following describes the tables and figures expected from SCORE studies, as well as approaches to analyzing the data and examples of SAS code.

Table 1. Baseline characteristics of participants, by arm.

This would include number of villages; number of participants; and age, sex, prevalence, and intensity assessed both by including all children and including only egg-positive children. Where appropriate, measures of dispersion should be included, for example, interquartile measures of intensity.

Figure 1. Map of study villages, by arm.

This can be presented as a single map, with different symbols for each of the arms, or as a series of maps.

Table 2. Coverage and harms.

Note that in Sm1 studies, only information on Arms 1-3 and treatment of SAC will be included.

		SAC treated	SAC total	% SAC treated	Total population treated	Total population eligible for treatment	% total population treated
**Year 1**							
	**Arm 1**						
	**Arm 2**						
	**Arm 3**						
	**Arm 4**						
	**Arm 5**						
	**Arm 6**						
**Year 2, etc.**							
	**Arm 1**						

In addition to the table, information should be provided about

- How numerators and denominators were collected, by year if they changed over time
- Any particular issues or concerns related to MDA coverage
- A listing of the serious adverse events or harms

Figure 2. Study flow by arm.

The study flow diagram describes the process through the phases of a randomized trial. A sample study flow diagram and accompanying table are shown in Appendix I.

Table 3. Descriptive results for baseline and Year 5.

	Arm 1 (cccc)	Arm 2 (ccss)	Arm 3 (cshh)	Arm 4 (ssss)	Arm 5 (sshh)	Arm 6 (shsh)
**No. tested at baseline**						
**No. infected at baseline**						
**Prevalence at baseline**						
**No. tested at Year 5**						
**No. infected at Year 5**						
**Prevalence at Year 5**						
**Absolute difference between prevalence at Year 5 and baseline**						
**Relative difference between prevalence at Year 5 and baseline (% change)**						
**Village-level arithmetic mean* infection intensity at baseline**						
**Village-level arithmetic mean* infection intensity at Year 5**						
**Egg reduction rate (1-Year 5 intensity/baseline)**						
**Individual-level arithmetic mean infection intensity at baseline***						
**Individual-level arithmetic mean infection intensity at Year 5***						

*Taken as the mean of all per village means per arm, not the mean of all individuals in the arm

Figure 3. Intensity categories, by Arm, by Year.

This is a stacked bar chart, showing low, medium, and high intensity infections, by Arm, for Years 1-5. Intensity categories are defined as:

- Sm: Low=1-99 epg, Medium=100-399 epg, high≥400 epg
- Sh: Low 1-50 eggs/10 ml, high≥51 eggs/10 ml

A supplemental table including the numbers used to make the chart should be available should modelers or other analysts want to use the exact data.

Table 4. Comparisons of prevalence and intensity at Year 5 among arms.

This table describes the primary outcomes.

	Unadjusted prevalence model estimate (CI)	Adjusted prevalence model estimate (CI)	Unadjusted intensity ratio (CI)	Adjusted intensity ratio (CI)
**Arm 1 v. Arm 4**				
**Arm 1 v. Arm 2**				
**Arm 1 v. Arm 3**				

**Arm 4 v. Arm 5**				
**Arm 4 v. Arm 6**				

General approach to analysis: The general approach is to use GEEs to estimate differences between the arms in year 5 only. We will report unadjusted estimates – with just village and arm fitted in the model – and adjusted estimates – where sex and age are also included in the model, along with weighting for number of children who provided data, because not all villages were able to sample 100 9-12 year old children.

ICC will be calculated using mixed models consistent with the GEE setup in the primary analysis.

All models will be based on individual level data on 9-12 year old children only.

Sample code: The following codes are provided as examples. Different studies may require further modification of the code below or may require different approaches.

<u>Unadjusted prevalence</u>

The evaluation of unadjusted prevalence uses a binomial GEE with logit link function. Village_ID is treated as the repeated subject, and we assume compound symmetry within a village. 'Lsmestimate' is used to test the pre-specified differences between arms.

```
ods trace off;
proc genmod data=allyears descending;
      where Study_Year = 5;
      class Study_Arm Village_ID/ param=glm;
      model Sh = Study_Arm / dist=bin link=logit ;
        repeated subject=Village_ID / type=cs;
      lsmeans Study_Arm / cl;
      lsmestimate Study_Arm "arm 2 vs. arm 1" -1 1 0 0 0 0 / cl;
      lsmestimate Study_Arm "arm 3 vs. arm 1" -1 0 1 0 0 0 / cl;
      lsmestimate Study_Arm "arm 4 vs. arm 1" -1 0 0 1 0 0 / cl;
      lsmestimate Study_Arm "arm 5 vs. arm 1" -1 0 0 0 1 0 / cl;
      lsmestimate Study_Arm "arm 6 vs. arm 1" -1 0 0 0 0 1 / cl;

      lsmestimate Study_Arm "arm 3 vs. arm 2" 0 -1 1 0 0 0 / cl;
      lsmestimate Study_Arm "arm 4 vs. arm 2" 0 -1 0 1 0 0 / cl;
      lsmestimate Study_Arm "arm 5 vs. arm 2" 0 -1 0 0 1 0 / cl;
      lsmestimate Study_Arm "arm 6 vs. arm 2" 0 -1 0 0 0 1 / cl;

      lsmestimate Study_Arm "arm 4 vs. arm 3" 0 0 -1 1 0 0 / cl;
      lsmestimate Study_Arm "arm 5 vs. arm 3" 0 0 -1 0 1 0 / cl;
      lsmestimate Study_Arm "arm 6 vs. arm 3" 0 0 -1 0 0 1 / cl;

      lsmestimate Study_Arm "arm 5 vs. arm 4" 0 0 0 -1 1 0 / cl;
      lsmestimate Study_Arm "arm 6 vs. arm 4" 0 0 0 -1 0 1 / cl;

      lsmestimate Study_Arm "arm 6 vs. arm 5" 0 0 0 0 -1 1 / cl;
estimate "O.R. arm 2 vs. arm 1" Study_Arm -1 1 0 0 0 0  / exp cl;
run;
```

Adjusted prevalence

The code is the same as for unadjusted prevalence, but with age, sex, and a weighting for village size added to the model.

villweight of individual = 1/# samples collected in village

```
ods trace off;
proc genmod data=allyears descending;
      where Study_Year = 5;
      class Study_Arm Village_ID Sex Age/ param=glm;
      model Sh = Study_Arm Sex Age / dist=bin link=logit ;
        repeated subject=Village_ID / type=cs;
      weight villweight ;
      lsmeans Study_Arm / cl;
run;
```

ICC of prevalence

```
/*macro*/
%macro icc_bin(data, cluster, outcome, out, m, stval=%str(), pred=%str());
proc nlmixed data=&data.;
      parms b0=0 &stval. var1=1;
      eta = exp(b0 + &pred. r);
      pred = eta / (1+eta);
      model &outcome. ~ binary(pred);
      random r ~ normal(0,var1) subject=&cluster.;
      estimate 'ICC' var1/(var1 + (constant('PI')**2)/3);
      estimate 'Deff' 1 + (&m.-1) * (var1/(var1 + (constant('PI')**2)/3));
      ods output AdditionalEstimates=&out.;
run;

data &out.;
      set &out.;
      cluster="&cluster.";
      outcome="&outcome.";
      m=&m.;
run;
%mend;

%icc_bin(sac, HHID, asc, hh1, 2.91, stval=%str(), pred=%str());
```

Unadjusted intensity

The code is the same as for unadjusted prevalence, but the distribution and link are changed.

```
ods trace off;
proc genmod data=allyears descending;
      where Study_Year = 5;
      class Study_Arm Village_ID/ param=glm;
      model Sh = Study_Arm / dist=nb link=log ;
        repeated subject=Village_ID / type=cs;
      lsmeans Study_Arm / cl;
run;
```

Adjusted intensity

The code is the same as for the adjusted prevalence, but the distribution and link are changed.

```
ods trace off;
proc genmod data=allyears descending;
      where Study_Year = 5;
      class Study_Arm Village_ID Sex Age/ param=glm;
      model Sh = Study_Arm Sex Age / dist=nb link=log ;
        repeated subject=Village_ID / type=cs;
      weight villweight ;
      lsmeans Study_Arm / cl;
run;
```

Figure 4. Village-level mean intensity by year, by arm.

Provide a line graph showing arithmetic mean intensity, by Year, by Arm.

VII. STUDY QUESTIONS AND END-POINTS OF INTEREST FOR COHORT STUDIES

a. Key research questions

The primary research questions in the cohort studies relate to comparisons of outcome measures between arms at Year 5, and the change from baseline and follow-up, by arm. The primary research questions are:

1a. Do morbidity markers among cohort children at Year 5 differ by study arm?

1b. How do changes in morbidity markers among cohort children from baseline to follow-up differ by arm?

Interpretation of the results of these analyses needs to be done in the context of the data on prevalence and intensity in the two arms. If data are available on malaria and other factors that could affect morbidity findings, these should be evaluated as well.

Further exploratory analysis should evaluate differences in morbidity by infection intensity. For example, in any given year, is there a relationship between intensity and morbidity measurements? Is there a relationship between changes in individual's intensity over time and changes in their morbidity measures? Note that some outcomes (e.g., liver pattern C) should be assessed taking cumulative exposure into account, whereas others are more likely to be related to contemporaneous exposure.

SCORE is committed to ensuring that the link between intensity and morbidity outcomes is evaluated. However, this is beyond the scope of the core SAP.

The original statistical analysis plans for evaluating the Cohort studies is found in Appendix J.

The following outcomes are of primary interest:

1.a. Height and/or weight, as calculated using WHO/CDC age-standardized Z-scores for height (HAZ), weight (WAZ) and body mass index (BAZ); these are typically treated as continuous outcomes.

1.b. Growth stunting (HAZ < -2) or nutritional wasting (BAZ < -2) as categorical outcomes.

2.a. Hb levels (continuous).

2.b. Anemia (categorical). Based on WHO criteria, anemia is defined as Hb < 11.5 g/dL for age < 12 years, Hb < 12 for females > 12 years, Hb < 12 for males 12-15, and Hb < 13 for males > 15. Altitude-related changes in normal circulating hemoglobin levels require adjustment of anemia cutoffs for higher elevations. The cutoffs are 0.2 gm/dL higher for each age group in Tanzania and Kenya locations as they are located at 1000-1250m elevation [1]

3. Maximum oxygen uptake, VO2 max (continuous) estimated based on shuttle run scores.
4. PedsQL scores (Total score and 4 subdomains: Psychosocial, Physical, School Performance, and Emotional) as continuous variables ranging from 0 to 100.
5. Liver patterns on abdominal ultrasound (categorical or ordinal scale).

Additional questions that could be explored relate to other ultrasound measures and whether the above measurements can be combined into a health and welfare score and be analyzed in regard to treatment arm.

b. Analyses, tables, and figures to be reported to SCORE from Cohort study researchers

The following describes the tables and figures expected from SCORE studies, as well as approaches to analyzing the data and examples of SAS code. Descriptive statistics such as mean and variance estimates, will be calculated. Linear or generalized linear models, which adjust for clustering effects, will be used to obtain parameters of interest, such as odds ratios (for binary outcome) and group-wise differences (continuous outcome), with their confidence limits. Confidence intervals will be based on empirical variance estimates corresponding to each of the models implemented, using sandwich estimators.

Table 1. Baseline and Year 5 characteristics of participants, and loss to follow-up, by arm.

This would include number of participants, age, sex, prevalence, and intensity assessed both by including all children and including only egg-positive children. Where appropriate, measures of dispersion should be included, for example, interquartile measures (IQR) or standard deviation (SD) of intensity.

	**Year 1**		**Year 5**		**Baseline characteristics of children lost to follow-up at Year 5**	
	**Arm 1** (cccc)	**Arm 6** (shsh)	**Arm 1**	**Arm 6**	**Arm 1**	**Arm 6**
**Number in cohort**						
**% female**						
**Age (SD)**						
**No. tested for schistosomiasis**						
**No. infected**						
**Prevalence**						
**Village-level arithmetic mean**						

**infection intensity***						
**Individual-level arithmetic mean infection intensity***						

*Taken as the mean of all per village means per arm, not the mean of all individuals in the arm

Table 2. Year 1/Year 5 Comparison of Prevalence and Intensity, by Arm

	**Year 1/Year 5 Comparison**					
	**Prevalence**		**Village-level intensity**		**Individual-level intensity**	
	**Arm 1**	**Arm 6**	**Arm 1**	**Arm 6**	**Arm 1**	**Arm 6**
**Absolute difference between Year 5 and baseline**						
**Relative difference between Year 5 and baseline (% change)**						
**Egg reduction rate (1-Year 5 intensity/baseline)**						

Figure 1. Study flow by arm. (See instructions for Figure 2 in the description of tables and figures for cross-sectional analysis.)

Table 3. Descriptive results on morbidity markers, by arm, by year.

	**Arm 1 (cccc)**			**Arm 6 (shsh)**		
	**Year 1**	**Year 3**	**Year 5**	**Year 1**	**Year 3**	**Year 5**
**Height: average Z score (SD)**						
**Weight: average Z score (SD)**						
**BMI: average Z score (SD)**						
**Prevalence of stunting % (CI)**						
**Prevalence of wasting %**						

**(CI)**						
**Mean hemoglobin in gm/dL (SD)**						
**Prevalence of anemia % (CI)**						
**Mean Vo2 max score (SD)**						
**Mean PedsQL score (SD)**<br>**Total Score**<br>**Physical**<br>**Emotional**<br>**Social**<br>**School**						
**Liver pattern**<br>**B % (CI)**<br>**C or higher % (CI)**						

Tables 4 (multiple tables). Comparison between Arm 1 and Arm 6 of Year 5 morbidity outcomes.

For each outcome, the following type of table should be developed. The results should be as indicated in the cells below.

Example: Table 4A. Unadjusted and adjusted models for **anemia** at Year 5.

	Unadjusted	Adjusted
**Arm (1 vs 6)**	Parameter of Interest** (CI)	Parameter of Interest (CI)
**Age**	---	Parameter of Interest (CI)
**Sex**	---	Parameter of Interest (CI)

General approach to analysis: For binary outcomes such as anemia status, the parameter of interest is typically the measure of association, such as odds ratio or relative risk. For models with other types of outcome, the parameter is the beta coefficient estimator from the corresponding models.

Generalized linear mixed effects models will be used to analyze the year 5 outcome. A generalized linear mixed effects model was chosen to account for random effects at the village level in year 5 comparison. The unadjusted model is a between-arm comparison without adjustment for other covariates. The adjusted model proposed here only includes adjustment for age and sex.

Generalized mixed effects models with normal, binomial, and multinomial distributional assumptions will be applied to address outcomes that are continuous, dichotomous, or categorical, respectively. SAS code examples for unadjusted models are provided below:

Sample code:

Linear mixed effects model code example

```
proc mixed;
where Study_Year=5;
class Person_ID Village_ID Study_Arm;
model y = Study_Arm / solution;
random intercept /subject=Village_ID;
run;
```

Binary outcome code example

```
proc glimmix;
where Study_Year=5;
class Person_ID Village_ID Study_Arm;
model y = Study_Arm / dist=binomial solution or;
random intercept /subject=Village_ID;
run;
```

Ordinal outcome code example

```
proc glimmix;
where Study_Year=5;
class Person_ID Village_ID Study_Arm y;
model y = Study_Arm / dist=multinomial solution;
random intercept /subject=Village_ID;
run;
```

Note that convergence could be an issue, and sometimes setting “method=LAPLACE” in the proc line could potentially help with the convergence.

Tables 5 (multiple tables). Comparison between Arm 1 and Arm 6 of change in morbidity outcomes from Year 1-Year 5.

For each outcome, the following type of table should be developed. The results should be as indicated in the cells below.

Example: Table 5A. Comparison of changes in **anemia** rates from Year 1 to Year 5 between Arms 1 and 6.

	**Unadjusted**	**Adjusted**
**Arm (1 vs. 6)**	Parameter of Interest (CI)	Parameter of Interest (CI)
**Year**	Parameter of Interest (CI)	Parameter of Interest (CI)
**Arm*Year**	Parameter of Interest (CI)	Parameter of Interest (CI)
**Age**	---	Parameter of Interest (CI)

**Sex**	---	Parameter of Interest (CI)

General approach to analysis: Generalized linear mixed effect models will be used to analyze the repeatedly measured outcomes from baseline to year 5. The mixed effects model was chosen to account for random effects at the village and individual levels from repeated measures.

Model setups are similar to those from the year 5 comparison (Table 7A), but with random effects from both the “Village_ID” and “Person_ID”. Changes from year 1 to year 5 between arms are modeled through the interaction term between “Study_Year” and “Study_Arm”. Models with normal, binomial, and multinomial distributional assumptions will be applied to address outcomes that are continuous, dichotomous, or categorical, respectively. SAS code examples for unadjusted models are provided below:

Sample code:

Linear mixed effects model code example

```
proc mixed;
class Person_ID Village_ID Study_Arm Study_Year(ref='1');
model y=Study_Year Study_Arm Study_Arm*Study_Year/solution;
random intercept /subject=Village_ID;
random intercept /subject=Person_ID(Village_ID);
run;
```

For binary variables

```
proc glimmix;
class Person_ID Village_ID Study_Arm Study_Year(ref='1');
model y=Study_Year Study_Arm Study_Arm*Study_Year/dist=binomial solution or;
random intercept /subject=Village_ID;
random intercept /subject=Person_ID(Village_ID);
run;
```

For ordinal outcomes

```
proc glimmix;
class Person_ID Village_ID Study_Arm Study_Year(ref='1') y;
model y=Study_Year Study_Arm Study_Arm*Study_Year/dist=multinomial solution;
random intercept /subject=Village_ID;
random intercept /subject=Person_ID(Village_ID);
run;
```

c. Missing cohort data

We acknowledge that large numbers of children were lost to follow-up over the five years of the cohort studies. We will not attempt to impute values for them.

Regarding missing outcome data for those included in the cohort, in the primary analyses only children who have all the data needed to assess a particular outcome will be analyzed. In a secondary analysis, multiple imputation and sensitivity analyses will be used to check the robustness of the complete case analysis for outcomes that have 10% or more children without the data needed to conduct the specific analysis. The investigators involved in the Kenya and Tanzania cohorts and SCORE will work together to determine the optimal way to address the missing data issue in their respective datasets.

Preliminary checks on missing data percentages at baseline were conducted for Sm cohort datasets. The missing percentages for major variables were below the 10% threshold, except for PedsQL (12.7%) and some ultrasound variables. Ultrasound variables that are missing in >=10% of children will not be included in any of the cohort analyses proposed in this SAP. Therefore, we anticipate that the small percentage of the analyses encountering missing data problems can be handled with multiple imputation. SAS programs used to check the missing data percentages are found in Appendix K: 'Additional SAS Code for Analysis of Cohort Data.'

The posterior distribution to be considered should reflect the noise associated with the uncertainty surrounding the parameters of the distribution that generates the data. Sensitivity checks will be conducted to ensure that the multiple imputation methods provide robust estimates. SAS codes and macros will be developed to conduct the multiple imputation and sensitivity analyses with pattern mixture models on the missing data. Some of the SAS programs developed for those purposes have already been included in the Appendix K: 'Additional SAS Code for Analysis of Cohort Data.'

Table 6. Comparison of imputed and unimputed values for outcomes with >10% missing data in Year 5 (from unadjusted models).

Outcome	Comparison Arm 1 vs. Arm 6 using raw data (Complete-Case Analysis)	Comparison Arm 1 vs. Arm 6 using multiple imputation
**Anemia**	Parameter of Interest (CI)	Parameter of Interest (CI)
**Weight**	Parameter of Interest (CI)	Parameter of Interest (CI)
…		

d. Additional SAS code

Additional SAS code that may be of use to individuals conducting analyses of cohort data are included in Appendix K.

VIII. PROTOCOL DEVIATIONS AND OTHER ISSUES

A full listing of known protocol deviations is in Appendix L. One important issue relates to an underlying premise in the original design of the Sm2 and Sh2 studies, i.e., that well-conducted CWT would yield higher overall SAC treatment coverage and would prove more effective than other approaches. In Kenya, however, the CWT intervention in Year 1 was less intense than in the SBT arms because schools were not

used as treatment venues by the study teams. This will be commented on in the discussion of results but not adjusted for.

A second important issue of concern is that testing and MDA were supposed to occur on an annual basis. In some study locations, the agreed-to testing and treatment schedule was not reliably followed. A variable has been created to flag villages in which this occurred.

Several issues have arisen during the collection of the SCORE datasets. These include:

- Limited enrollment in some villages: The target study population size was 100 9-12 year old children. In some villages, the enrollment was significantly less. To account for this, a "village-weight" term will be added to the GEE model to weight results according to numbers of children tested per village.
- Truncation of Kato-Katz egg counts: In Kenya Sm1 and Sm2, egg counts were truncated at 42 eggs per slide in Years 1-3; they were truncated at 1,000 subsequently, which is more consistent with the laboratory practice used in other Sm clinical studies. We will not attempt to model the "true" egg counts for children with results recorded at the truncation level. We will present results indicating the recorded egg values and calculated epg values, and also categorized as no infection, and low, medium, or high-intensity infections.
- Missing data on some children
    - Children without data on age, sex, and presence or absence of eggs on at least one slide were not included in the study, with the exception of Mozambique, where 52 children (.9% of the total tested) are missing sex data in Year 1, and a smaller number are missing such data in later years. Children with missing sex data in Mozambique are largely from one village.
    - Issues related to some children having fewer slides than required by the protocol have been discussed elsewhere.
- Missing data are an issue for a limited number of variables in the cohort. This is discussed previously.

REFERENCES

Campbell MK, Piaggio G, Elbourne DR, Altman DG, et al. Consort 2010 statement: extension to cluster randomized trials. BMJ 2012;345:e5661.

WHO/CDC. Assessing the iron status of populations: report of a Joint World Health Organization/Centers for Disease Control and Prevention. Technical consultation on the assessment of iron status at the population level. Geneva, Switzerland, 6–8 April 2004. 2e ed. Geneva: World Health Organization; 2007.

APPENDICES

A. Harmonized Protocol for SCORE Gaining and Sustaining Control Studies
B. Harmonized Protocol for SCORE Cohort Studies of Subtle Morbidity
C. Definitions for SCORE Studies
D. Data Elements Available for Analysis
E. Data Dictionary for SCORE Gaining and Sustaining Control Studies
F. Data Dictionary for SCORE Cohort Studies
G. Data Dictionary for Coverage
H. Initial Analysis Plan for SCORE Gaining and Sustaining Control Studies
I. Example of a Study Flow Diagram
J. Initial Analysis Plan for SCORE Cohort Studies of Subtle Morbidities
K. Additional SAS Code for Analysis of Cohort Data
L. Protocol Deviations

Supplemental Material
Statistical Analysis Plan (SAP) for
SCORE Studies of Annual and Biannual MDA for *S. haematobium* in Niger

Version 1.0, 3 January 2017

Authors: the SAP-Niger Working Group

PREFACE

This statistical analysis plan (SAP) has been developed by SCORE team members to standardize the evaluation of outcomes data from SCORE urogenital schistosomiasis control studies in Niger. It provides guidance on appropriate data sources, variable definitions, and approaches for assessing quantitative outcomes. The intent is to provide consistent analysis of implementation and outcomes data for each arm of the study, and for the study overall. The data analysis plan presented below and in the Appendices is based on statistical methods that are available using SAS. Use of other statistical analysis packages is also appropriate.

The contents of this SAP represent a minimum analysis for this study. The tables described herein should be completed and provided to SCORE before the conclusion of the study for SCORE to use in reporting to the Bill & Melinda Gates Foundation (BMGF). They should be available for sharing upon appropriate request, e.g., as supplemental tables for publications. The choice of data to present in published papers is left to authors and will reflect country-specific needs and interests; however, the definitions and approaches described in this document should be used in analysis and published results should be consistent with those from the SAP analyses. In addition, investigators should review the guidelines from CONSORT (Campbell 2012) to ensure that they address all requirements for reporting randomized trials. Additional exploratory analyses beyond those described here are encouraged.

Table of Contents

PREFACE 1

I. Study Abbreviations and Brief Definitions 2

II. GOALS AND OBJECTIVES OF THE SCORE-ANNUAL VS. BIANNUAL S. HAEMATOBIUM TREATMENT STUDIES 3

a. Overview of SCORE-Niger MDA studies 3

b. Study design: Original design and the revised 2013 protocol 3

c. Limitations to the analyses included in this SAP 8

III. DATA COLLECTION, AND TYPES OF DATA AVAILABLE 8

a. Individual-level data 8

b. Village-level data 8

IV. SCORE UNIFORM DATASETS 9

V. DEFINITIONS OF PREVALENCE, INTENSITY, AND COVERAGE 9
VI. STUDY QUESTIONS AND END-POINTS OF INTEREST FOR THE SCORE-NIGER STUDY 10
a. Key research questions 10
b. Analyses, tables, and figures to be reported to SCORE by the SCORE-Niger Study researchers........ 10
Table 1. Baseline characteristics of participants, by arm. 10
Figure 1. Map of study villages, by arm. 10
Table 2. Coverage and harms. 11
Figure 2. Study flow by arm. 11
Table 3. Descriptive results for baseline and Year 5. 12
Figure 3. Intensity categories, by Arm, by Year. 12
Table 4. Comparisons of prevalence and intensity at Year 5 among arms. 13
Figure 4. Village-level mean intensity by year, by arm. 15
VII. PROTOCOL DEVIATIONS AND OTHER ISSUES 15
REFERENCES 15
APPENDICES 16
A. Harmonized Protocol for SCORE Gaining and Sustaining Control Studies 16
B. Harmonized Protocol for SCORE Cohort Studies of Subtle Morbidity 16
C. Protocol for the Redesigned Niger Study 16
D. Data Elements Available for Analysis 16
E. Data Dictionary for SCORE Gaining and Sustaining Control Studies 16
F. Data Dictionary for SCORE Coverage 16
G. Example of a Study Flow Diagram 16
H. Protocol Deviations 16

I. STUDY ABBREVIATIONS AND BRIEF DEFINITIONS

CWT – Community-wide treatment
GEE - Generalized estimating equations
ICC – intra-class correlation
MDA – Mass drug administration delivered on an annual basis at a target dose of 40 mg/kg, with dose estimated from subject height using a dosing pole
SAC – School age children
SBT – School-based treatment
Sh – *Schistosoma haematobium*

SCORE — Schistosomiasis Consortium for Operational Research and Evaluation
SUDS – SCORE Uniform Data Set
Y – Year: Y1 data collection is defined as the baseline data collection, which occurs before treatment. Y1 treatment refers to the first MDA treatment

Abbreviations related to study arm treatment sequences
c – CWT
h- holiday
s – SBT

II. GOALS AND OBJECTIVES OF THE SCORE-ANNUAL VS. BIANNUAL *S. HAEMATOBIUM* TREATMENT STUDIES

The overall goal of the SCORE project is to provide an evidence base and tools for programmatic decisions on how best to extend control of *Schistosoma* infections to achieve morbidity control, and, eventually, elimination. The SCORE-Gaining and Sustaining Control and Cohort studies are randomized trials designed to inform approaches to mass drug administration (MDA). These were developed through a collaborative process. The original harmonized protocols, which were to be followed by all groups funded to conduct this work, appear in Appendices A (Gaining and sustaining studies) and B (Cohort studies). Niger was to conduct a gaining, a sustaining, and a cohort study. A Standardized Analysis Plan (SAP) for these studies is available from SCORE upon request. However, because Niger did not follow the study protocol, the cohort study was discontinued, and the Niger study was redesigned in Year 3. The protocol for the redesigned Niger study is in Appendix C and a map of the redesigned study in in appendix X. This SAP has been developed for the redesigned Niger study.

a. Overview of SCORE-Niger MDA studies

Niger was funded to conduct studies of urogenital schistosomiasis. Sh1 studies involved communities with prevalence during eligibility testing of <24%, and Sh2 studies involved communities with eligibility prevalence of $\geq$24%.

The original design of the Niger Sh1 and Sh2 gaining and sustaining control studies was predicated on the idea that randomization would result in starting prevalences being roughly equivalent in different study arms. However, because of the way in which Niger randomized its villages – with geographic clustering, starting prevalences were markedly different in the different arms, so that valid comparisons between arms could not be made.

b. Study design: Original design and the revised 2013 protocol

Intended design: The Gaining and Sustaining studies are parallel cluster-randomized, open-label operational research trials of PZQ MDA for control of prevalence and intensity of *Schistosoma* infections in endemic communities in Africa.

For purposes of this protocol, a study community or village was required to have a primary school, because several arms of the study are school-based and every participating community must be eligible to be randomized to any of the study arms. However, a study community could have more than one school. If two nearby communities had schools with fewer than 100 children per school, but they were similar, they could be combined for purposes of this study and be considered as one study community.

Where schools had fewer than 100 schoolchildren aged 9-12 years, additional non-attending school aged children were recruited from the local villages. In the case of villages that shared water sources, one village was selected randomly to participate.

Prior to enrollment, village eligibility for either Sh1 or Sh2 in the original study design was determined by testing fifty 13-14 year-old children in each village. Once a village was enrolled in the study, the **primary outcomes of interest are prevalence and intensity among 9-12 year-old children**. A simple randomization procedure was used, without stratification. Re-randomization based on the first randomization results was not permitted.

In studies of communities with lower levels of *S. haematobium* prevalence (<24%, Sh1 studies), only SBT was implemented on an annual or every other year basis. The *original* study Sh1 had three desired study arms arranged as follows:

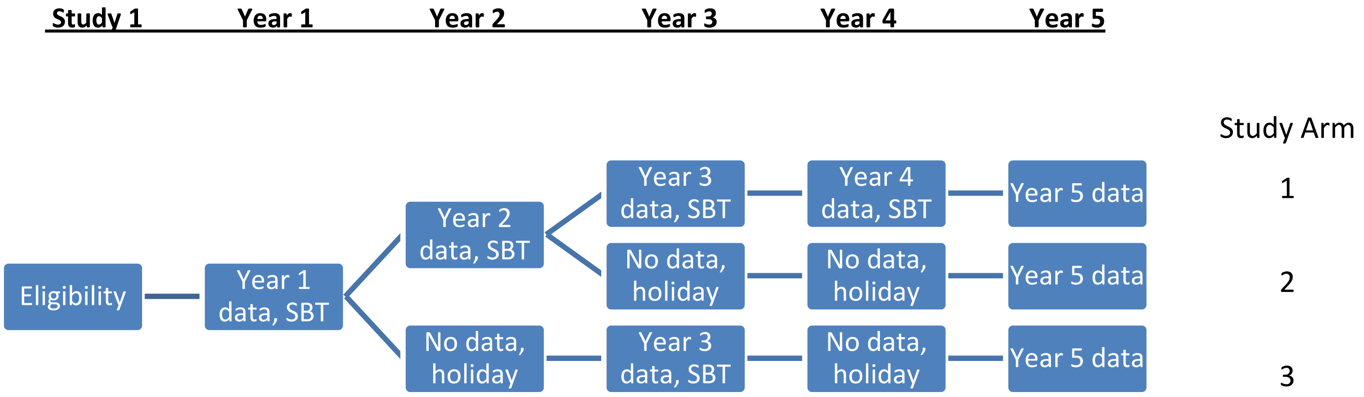

In studies of communities with higher levels of *S. haematobium* prevalence (≥ 24 %, Sh2 studies), either CWT or SBT was implemented on an annual or every other year basis. The *original* Study Sh2 had six desired study arms arranged as follows:

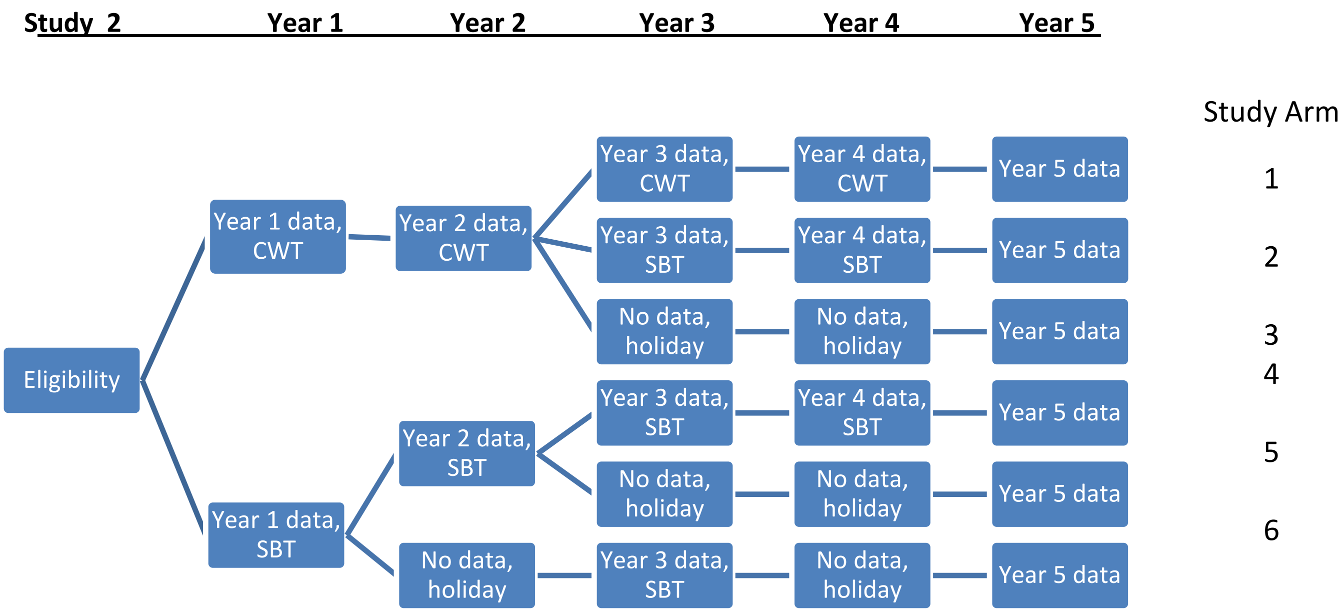

Populations and expected sample size: The goal of the study was to enroll 25 eligible villages per study arm, and to monitor each village's prevalence and intensity of *Schistosoma haematobium* infection among **a random sample of 100 resident 9-12 year-old children each year**, starting before implementation of MDA (Year 1) and continuing through Year 5 (see study arm schema above). Nominal enrollment for the Sh1 study was 75 villages, and for the Sh2 study, 150 villages, with to 10,000-22,500 children tested each year, according to schedule. (**N.B.** Communities assigned to 'holiday' were not tested in that year.) Because of the way in which Niger randomized its villages – with geographic clustering, starting prevalence was markedly different, so that valid comparisons between arms could not be made. Therefore, **the Niger study was re-designed.**

The failure to randomize appropriately was recognized following Y2 data collection, after two years of MDA. The current protocol is a BMGF-approved replacement for the previously approved protocol for gaining and sustaining studies. Within the following revised arms, randomization was carefully done at the *community,* not regional, level. The new study objective was to evaluate the impact of twice-a-year vs. once-a-year treatment with PZQ. Once-a-year and twice-a-year MDA, given in Years 3 and 4 of each study, were compared in the context of communitywide treatment (CWT) or school-based treatment (SBT) according the following revised schema:

- The previous Sh2 study Arms 1-3 are receiving CWT for the remainder of the study, starting with the 2013 MDA.
- Arms 4-6 from Study Sh2 and arms 1-3 from Study Sh1 from the original study design are receiving school-based treatment (SBT) for the remainder of the study, starting with the 2013 MDA.
- Within each arm, villages were randomized to receive either once or twice yearly treatment. Those with twice-yearly treatment will receive two MDA treatments during 2013 in June and December and two in 2014 in June and December.

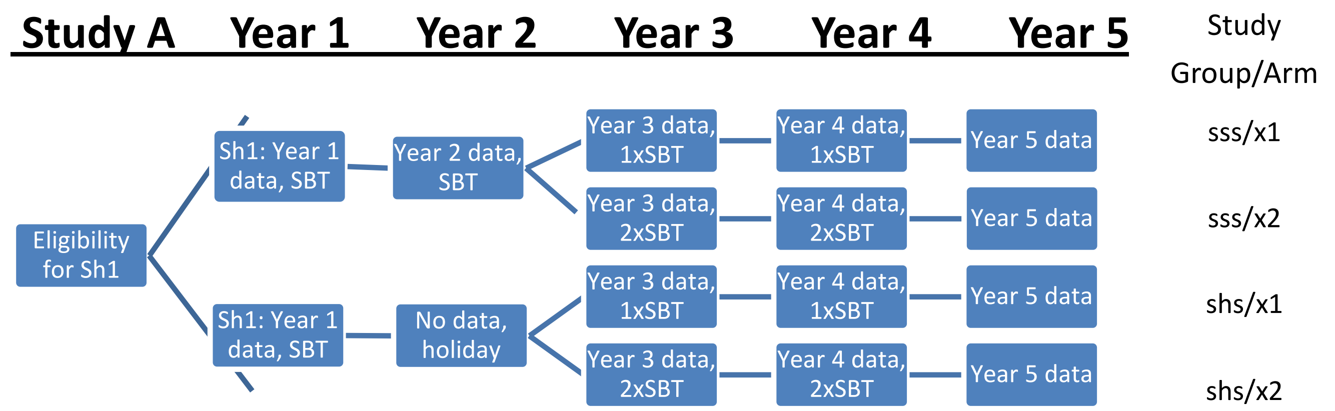

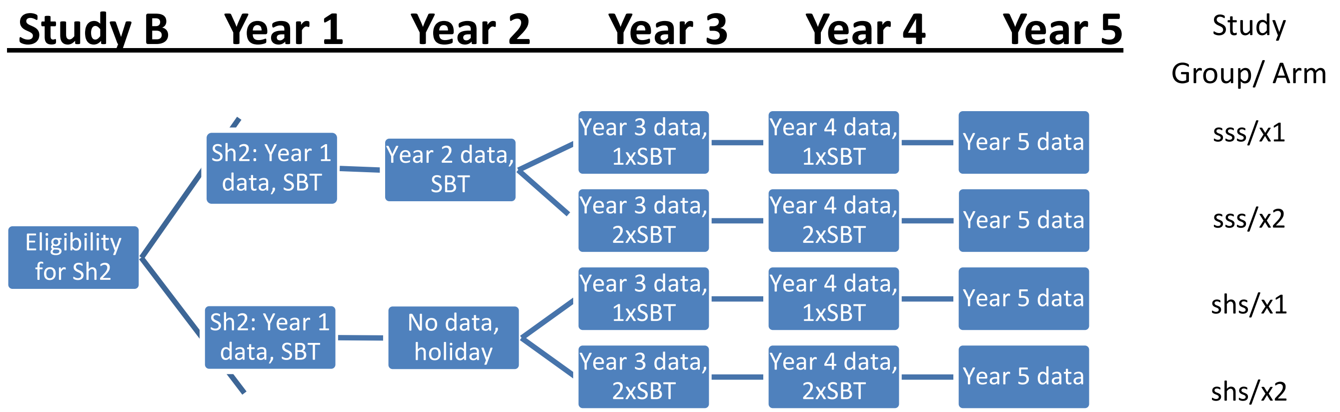

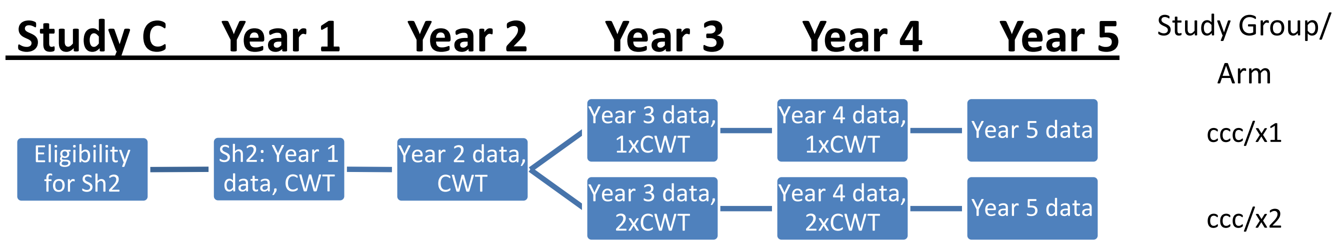

In addition to 9-12 year-old children, SCORE studies included some data collection on first-year students and in adults. More information about data collected from these populations is provided in the protocols

and in Appendix D. Their results are not considered in this SAP, but may be used for future secondary analyses of the SCORE gaining and sustaining studies.

c. Limitations to the analyses included in this SAP

- Adverse events during MDAs were recorded by those administering the drugs in a manner consistent with WHO or country guidelines. These records were not requested as part of the SCORE research. Therefore, the planned SAP analyses do not include a "safety" component.
- Cost data collected in Year 2 were also impacted by the failure to randomize appropriately and are not considered further.

III. DATA COLLECTION, AND TYPES OF DATA AVAILABLE

The table in Appendix D describes the data elements available from the SCORE-Niger Study.

a. Individual-level data

Parasitological data on *Schistosoma* infection among children ages 9-12 were collected prior to each MDA and a year following the last treatment.

In the Sh control studies, 10 ml of urine from a single well-stirred urine sample for each individual was filtered twice, and each of these two filtrations was examined under the microscope; the number of eggs found in each slide and the corresponding volume of urine filtered was recorded.

Parasitological data were also collected in Years 1, 3, and 5 on children in the first-year class and in Years 1 and 5 on adults (see Appendix D). However, given that data on these populations do not represent the primary end-points of the SCORE treatment studies, and that the data collected on them are much more limited than that for 9-12 year olds, <u>they will not be reported as primary end-points</u>.

b. Village-level data

Village-level data were collected about usual water sources, sanitation, and other issues by key-informant interviews. Coverage data were collected following each MDA. In general, the data on numbers receiving treatment was provided by those administering treatment – either school personnel or community health workers. The national program administered the MDA. Denominator data came from a governmental census reports that were collected from the local health centers responsible for coordinating the MDA.

Coverage values for both SAC and the entire population were requested. Coverage was calculated using the governmental denominator and treatment numbers reported by the national program.

Data are also available on snail quantities, species, and schistosome population genetics from selected communities in the Niger studies.

IV. SCORE UNIFORM DATASETS

SCORE used standardized approaches to data cleaning and creation of analytic data sets. Data were cleaned in country based on a series of checks provided by SCORE. "Clean" data were sent to SCORE for further assessment, and issues identified with the data (e.g., missing data, data that appeared to be out of range, etc.) were sent back to the country to be corrected to the extent possible.

Rules employed in the SUDS datasets include:

- Individuals who did not have parasitological data recorded were entirely excluded from the datasets.
- Inclusion in the age groups: 5-8 years, 9-12 years, and adult was based on recoded ages. If the recorded age did not meet study criteria, these were classified as "Other."

Once clean, data were placed in the SCORE Uniform Data Set (SUDS) format, and returned to the country. Datasets for each study are stored separately.

Each data set is available in SAS, Excel, and *.csv formats, and is accompanied by the appropriate SUDS data dictionary. The data dictionary also includes metadata pages specific for each study site, indicating issues identified during data collection and cleaning that may need to be taken into account in analysis.

SUDS data dictionaries for Sh studies are in Appendix E. The SUDS data dictionary for coverage is in Appendix F.

V. DEFINITIONS OF PREVALENCE, INTENSITY, AND COVERAGE

The following definitions will be used for individual-level data:

- Individual mean eggs per 10 ml (Sh studies): Number of eggs found * 10 / volume of urine filtered. If estimated counts are over 1,000 after volume adjustment, they should be truncated at 1,000. Mean eggs per 10 ml will be used for both analysis and reporting, as this measure is both continuous and a standard metric. Note that some software packages may require mean eggs per 10 ml to be rounded prior to analysis, but this will only affect individuals with less than 10ml of urine filtered.
- Egg positive: a child will be deemed to be egg-positive if one or more eggs were found in any of the slides examined.

The following definitions will be used for reporting on cross-sectional studies:

- **Prevalence of *Schistosoma* infection:** Percentage of egg-positive children among the 9-12 year olds tested in each community each year.
- **Mean intensity of *Schistosoma* infection:** *Arithmetic* mean of individual mean eggs per 10 ml among the 9-12 year-old children. Two values are to be reported:
    - i) <u>Village-level intensity</u>: This is the mean egg count for all tested 9-12 year-old subjects (including those with zero egg counts), which is a measure of community-level contamination potential.
    - ii) <u>Individual-level intensity</u>: This is the mean egg count among egg-positive subjects, which is an estimate of the intensity of infection among known active cases.

- **SBT coverage:** Numbers of school-age children treated / numbers of school-age children.
- **CWT coverage**: Numbers of people in the community treated / number of treatment-eligible people in the community.

VI. STUDY QUESTIONS AND END-POINTS OF INTEREST FOR THE SCORE-NIGER STUDY

The following study questions and endpoints of interest are derived directly from the objectives as stated in the original, harmonized SCORE-Niger protocol.

a. Key research questions

The primary research questions are:

1a. Does the final (Year 5) prevalence of schistosomiasis **among children age 9-12** differ by treatment frequency in years 3 and 4?

1b. Does the final (Year 5) mean intensity of Sh infection among **children aged 9-12** differ by treatment frequency in years 3 and 4?

More specifically, the planned analysis will involve a single arm-to-arm comparison in each study. We will not combine the studies as they were in different geographical areas and have different treatment histories.

b. Analyses, tables, and figures to be reported to SCORE by the SCORE-Niger Study researchers

The following describes the tables and figures expected from SCORE studies, as well as approaches to analyzing the data and examples of SAS code.

Table 1. Baseline characteristics of participants, by arm.

This would include number of villages; number of participants; and age, sex, prevalence, and intensity assessed both by including all children and including only egg-positive children. Where appropriate, measures of dispersion should be included, for example, interquartile measures of intensity.

Figure 1. Map of study villages, by arm.

This can be presented as a single map, with different symbols for each of the arms, or as a series of maps.

Table 2. Coverage and harms.

**Study A**	**SAC treated**	**SAC total**	**% SAC treated**	**Total population treated**	**Total population eligible for treatment**	**% total population treated**
**Year 1**						
**sss/ x1 1**						
**sss/ x2**						
**shs/ x1**						
**shs/ x2**						
**x1 combined**						
**x2 combined**						
**Year 2**						
**sss/ x1**						
**sss/ x2**						
**shs/ x1**						
**shs/ x2**						
**x1 combined**						
**x2 combined**						
**Year 3**						
**[…]**						

A separate table should be constructed for each of study A, B & C. The figures for arms 1 & 2 in grey above should be the total (or average) of the relevant figures from each of the separate Sh1/Sh2 groups above.

In addition to the table, information should be provided about

- How numerators and denominators were collected, by year if they changed over time
- Any particular issues or concerns related to MDA coverage
- A listing of the serious adverse events or harms

Figure 2. Study flow by arm.

The study flow diagram describes the process through the phases of a randomized trial. A sample study flow diagram and accompanying table are shown in Appendix G.

Table 3. Descriptive results for baseline and Year 5.

**Study A**	**sss/ x1**	**sss/ x2**	**shs/ x1**	**shs/ x2**	**x1 combined**	**x2 combined**
**No. tested at baseline**						
**No. infected at baseline**						
**Prevalence at baseline**						
**No. tested at Year 5**						
**No. infected at Year 5**						
**Prevalence at Year 5**						
**Absolute difference between prevalence at Year 5 and baseline**						
**Relative difference between prevalence at Year 5 and baseline (% change)**						
**Village-level arithmetic mean infection intensity at baseline**						
**Village-level arithmetic mean infection intensity at Year 5**						
**Egg reduction rate (1-Year 5 intensity/baseline)**						
**Individual-level arithmetic mean infection intensity at baseline**						
**Individual-level arithmetic mean infection intensity at Year 5**						

A separate table should be constructed for each of study A, B & C. The figures for arms 1 & 2 in grey above should be the total (or average) of the relevant figures from each of the separate Sh1/Sh2 groups to the left.

Figure 3. Intensity categories, by Arm, by Year.

This is a stacked bar chart, showing low, medium, and high intensity infections, by Arm, for Years 1-5, and each study separately. Intensity categories are defined as:

- Sh: Low 1-50 eggs/10 ml, high$\geq$51 eggs/10 ml

A supplemental table including the numbers used to make the chart should be available should modelers or other analysts want to use the exact data.

Table 4. Comparisons of prevalence and intensity at Year 5 among arms.

This table describes the primary outcomes.

	Unadjusted prevalence model estimate (CI)	Adjusted prevalence model estimate (CI)	Unadjusted intensity ratio (CI)	Adjusted intensity ratio (CI)
**Study A: Arm 1 v. Arm 2**				
**Study B: Arm 1 v. Arm 2**				
**Study C: Arm 1 v. Arm 2**				

General approach to analysis: The general approach is to use mixed models to estimate differences between the arms in year 5 only, analyzing each study separately. We will report unadjusted estimates – with just village, study group and arm fitted in the model – and adjusted estimates – where sex and age are also included in the model. The mixed model will implicitly weight for any villages that were not able to sample 100 9-12 year old children.

ICC will be calculated using mixed models in the primary analysis.

All models will be based on individual level data on 9-12 year old children only.

Sample code: The following codes are provided as examples.

Unadjusted prevalence

The evaluation of unadjusted prevalence uses a binomial mixed model with logit link function. Village ID is treated as the repeated subject, and we assume compound symmetry within a village. 'Lsmestimate' is used to test the pre-specified differences between arms.

```
ods trace off;
proc mixed data=allyears descending;
      where Study_Year = 5;
      class Study_Arm Village_ID Study_Group/ param=glm;
      model Sh = Study_Arm + Study_Group / dist=bin link=logit;
        random=Village_ID / type=cs;
      lsmeans Study_Arm / cl;
run;
```

Adjusted prevalence

The code is the same as for unadjusted prevalence, but with age, sex, and a weighting for village size added to the model. Study_Arm and Study_Group should be tested for – if found to be significant, comparisons between arms should be reported separately for each study.

villweight of individual = 1/# samples collected in village

```
ods trace off;
proc mixed data=allyears descending;
      where Study_Year = 5;
      class Study_Arm Village_ID Group Sex Age/ param=glm;
      model Sh = Study_Arm Group Sex Age / dist=bin link=logit ;
        random=Village_ID / type=cs;
      lsmeans Study_Arm / cl;
run;
```

ICC of prevalence

```
/*macro*/
%macro icc_bin(data, cluster, outcome, out, m, stval=%str(), pred=%str());
proc nlmixed data=&data.;
      parms b0=0 &stval. var1=1;
      eta = exp(b0 + &pred. r);
      pred = eta / (1+eta);
      model &outcome. ~ binary(pred);
      random r ~ normal(0,var1) subject=&cluster.;
      estimate 'ICC' var1/(var1 + (constant('PI')**2)/3);
      estimate 'Deff' 1 + (&m.-1) * (var1/(var1 + (constant('PI')**2)/3));
      ods output AdditionalEstimates=&out.;
run;

data &out.;
      set &out.;
      cluster="&cluster.";
      outcome="&outcome.";
      m=&m.;
run;
%mend;

%icc_bin(sac, HHID, asc, hh1, 2.91, stval=%str(), pred=%str());
```

Unadjusted intensity

The code is the same as for unadjusted prevalence, but the distribution and link are changed. Study_Arm and Study_Group should be tested for – if found to be significant, comparisons between arms should be reported separately for each study.

```
proc mixed data=allyears descending;
      where Study_Year = 5;
      class Study_Arm Village_ID Study_Group/ param=glm;
      model Sh = Study_Arm Study_Group / dist=nb link=log;
        random=Village_ID / type=cs;
      lsmeans Study_Arm / cl;
run;
```

Adjusted intensity

The code is the same as for the adjusted prevalence, but the distribution and link are changed. Study_Arm and Study_Group should be tested for – if found to be significant, comparisons between arms should be reported separately for each study.

```
ods trace off;
proc mixed data=allyears descending;
      where Study_Year = 5;
      class Study_Arm Village_ID Sex Age/ param=glm;
      model Sh = Study_Arm Study_Group Sex Age / dist=nb link=log ;
        random=Village_ID / type=cs;
      lsmeans Study_Arm / cl;
run;
```

Figure 4. Village-level mean intensity by year, by arm.

Provide a line graph showing arithmetic mean intensity, by Year, by Arm.

VII. PROTOCOL DEVIATIONS AND OTHER ISSUES

A full listing of known protocol deviations is in Appendix H.

In addition to the failure to randomize at the village level in Niger, several issues have arisen during the collection of the SCORE datasets. These include:

- Limited enrollment in some villages: The target study population size was 100 9-12 year old children. In some villages, the enrollment was significantly less. To account for this, a “village-weight” term will be added to the GEE model to weight results according to numbers of children tested per village.
- Missing data on some children. Note that children without data on age, sex, and presence or absence of eggs on at least one slide were not included in the study.

REFERENCES

Campbell MK, Piaggio G, Elbourne DR, Altman DG, et al. Consort 2010 statement: extension to cluster randomized trials. BMJ 2012;345:e5661.

APPENDICES

A. Harmonized Protocol for SCORE Gaining and Sustaining Control Studies
B. Harmonized Protocol for SCORE Cohort Studies of Subtle Morbidity
C. Protocol for the Redesigned Niger Study
D. Data Elements Available for Analysis
E. Data Dictionary for SCORE Gaining and Sustaining Control Studies
F. Data Dictionary for SCORE Coverage
G. Example of a Study Flow Diagram
H. Protocol Deviations

Supplemental Table S1: SCORE's Kenya Gaining Control Study comparisons of *S. mansoni* infection prevalence and intensity among 9-12 year-old children between study arms at Year 5, adjusted for community level clustering effects, age, and sex

	**Prevalence Ratio**		**Intensity Ratio**	
**Study Intervention Comparison**	**Adjusted PR (CI)**	**p**	**Adjusted AMR (CI)**	**p**
CCSS vs. CCCC	0.59 (0.34, 1.03)	0.06	0.36 (0.10, 1.24)	0.11
CCHH vs. CCCC	1.19 (0.76, 1.87)	0.44	0.88 (0.36, 2.18)	0.78
SSSS vs. CCCC	0.73 (0.42, 1.28)	0.28	0.48 (0.18, 1.30)	0.15
SSHH vs. CCCC	1.09 (0.69, 1.73)	0.71	0.99 (0.34, 2.87)	0.99
SHSH vs. CCCC	1.23 (0.78, 1.95)	0.37	1.01 (0.34, 3.00)	0.99
**CCHH vs. CCSS**	**2.03 (1.23, 3.34)**	**0.005**	2.44 (0.82, 7.27)	0.11
SSSS vs. CCSS	1.25 (0.68, 2.27)	0.47	1.34 (0.42, 4.27)	0.62
**SSHH vs. CCSS**	**1.85 (1.11, 3.09)**	**0.02**	2.75 (0.81, 9.35)	0.10
**SHSH vs. CCSS**	**2.09 (1.26, 3.47)**	**0.004**	2.80 (0.80, 9.75)	0.11
SSSS vs. CCHH	0.61 (0.37, 1.01)	0.06	0.55 (0.25, 1.21)	0.14
SSHH vs. CCHH	0.91 (0.62, 1.35)	0.65	1.13 (0.46, 2.73)	0.79
SHSH vs. CCHH	1.03 (0.70, 1.51)	0.88	1.15 (0.46, 2.86)	0.77
SSHH vs. SSSS	1.49 (0.89, 2.49)	0.13	2.05 (0.78, 5.39)	0.15
**SHSH vs. SSSS**	**1.68 (1.01, 2.80)**	**0.045**	2.08 (0.77, 5.63)	0.15
SHSH vs. SSHH	1.13 (0.76, 1.68)	0.55	1.02 (0.35, 2.97)	0.98

Abbreviations: AMR, arithmetic mean ratio; C, communitywide treatment; H, praziquantel holiday – a year when no praziquantel MDA was provided; PR, prevalence ratio; S, school-based treatment. **Bold** font indicates statistically significant differences.

Supplemental Table S2: SCORE's Tanzania Gaining Control Study comparisons of *S. mansoni* infection prevalence and intensity among 9-12 year-old children between study arms at Year 5, adjusted for community level clustering effects, age, and sex

**Study Intervention Comparison**	**Adjusted prevalence ratio (95% CI)**	**Adjusted intensity ratio (95% CI)**
SSSS vs. CCCC	1.09 (0.55, 2.16)	0.96 (0.48, 1.91)
CCSS vs. CCCC	1.05 (0.55, 2.00)	1.04 (0.51, 2.12)
CCHH vs. CCCC	1.45 (0.80, 2.69)	1.38 (0.70, 2.72)
SSHH vs. SSSS	1.33 (0.64, 3.01)	1.91 (0.97, 3.76)
SHSH vs. SSSS	1.31 (0.63, 2.71)	1.30 (0.61, 2.79)

Abbreviations: C, communitywide treatment; H, praziquantel holiday – a year when no praziquantel MDA was provided; S, school-based treatment.

Supplemental Table S3: SCORE's Mozambique Gaining Control Study comparisons of *S. haematobium* infection prevalence and intensity among 9-12 year-old children between study arms at Year 5, adjusted for community level clustering effects, age, and sex

**Study Intervention Comparison**	**Adjusted prevalence ratio (95% CI)**	**Adjusted intensity ratio (95% CI)**
SSSS vs. CCCC	1.01 (0.62, 1.69)	1.62 (0.74, 3.21)
CCSS vs. CCCC	1.08 (0.69, 1.69)	1.56 (0.76, 3.21)
**CCHH vs. CCCC**	1.45 (0.85, 2.47)	**2.15 (1.10, 4.18)**
**SSHH vs. SSSS**	**1.73 (1.08, 2.77)**	1.32 (0.66, 2.61)
SHSH vs. SSSS	1.12 (0.62, 2.02)	0.70 (0.32, 1.53)

Abbreviations: C, communitywide treatment; H, praziquantel holiday – a year when no praziquantel MDA was provided; S, school-based treatment.
Bold font indicates statistically significant differences.

Supplemental Table S4: SCORE's Kenya Sustaining Control Study comparisons of *S. mansoni* infection prevalence and intensity among 9-12 year-old children between study arms at Year 5, adjusted for community level clustering effects, age, and sex

	**Prevalence Ratio**		**Intensity Ratio**	
**Study Intervention Comparison**	**Adjusted PR (CI)**	**P**	**Adjusted AMR (CI)**	**p**
SSHH vs. SSSS	1.49 (0.92, 2.39)	0.10	1.62 (0.83, 3.15)	0.16
SHSH vs. SSSS	1.10 (0.72, 1.67)	0.66	0.86 (0.47, 1.60)	0.64
**SHSH vs. SSHH**	0.74 (0.48, 1.13)	0.17	**0.53 (0.29, 0.98)**	**0.04**

Abbreviations: AMR, arithmetic mean ratio; H, praziquantel holiday – a year when no praziquantel MDA was provided; PR, prevalence ratio; S, school-based treatment.
Bold font indicates statistically significant differences.

Supplemental Table S5: SCORE's Côte d'Ivoire Sustaining Control Study comparisons of *S. mansoni* infection prevalence and intensity among 9-12 year-old children between study arms at Year 5, adjusted for community level clustering effects, age, and sex

	**Prevalence Ratio**	**Intensity Ratio**
**Study Intervention Comparison**	**Adjusted PR (CI)**	**Adjusted AMR (CI)**
SSHH vs. SSSS	1.96 (0.88, 4.35)	1.41 (0.41, 5.0)
SHSH vs. SSSS	1.85 (0.78, 4.35)	1.96 (0.55, 7.14)
SHSH vs. SSHH	0.94 (0.58, 1.54)	1.39 (0.85, 2.27)

Abbreviations: AMR, arithmetic mean ratio; H, praziquantel holiday, a year when no praziquantel MDA was provided; PR, prevalence ratio; S, school-based treatment.

Supplemental Table S6: Results for the modified Niger MDA trial, in which communities were randomized to receive once yearly or twice yearly praziquantel MDA for *S. haematobium* infection following an initial one or two rounds of SBT or CWT. Group A includes communities with microhematuria prevalence in eligibility testing of 5-20%. Groups B and C had prevalence in eligibility testing of >20%. See Figure 2 of the paper for study design details.

	Group A						Group B						Group C	
	SSSS x1	SSSS x 2	SHSS x1	SHSS x2	**A1**	**A2**	SSSS x1	SSSS x2	SHSS x1	SHSS x2	**B1**	**B2**	**CCCC x1**	**CCCC x2**
No. tested at baseline	2,309	2,226	1,152	995	**3,461**	**3,221**	2,448	2,401	1,211	1,270	**3,659**	**3,671**	**3,357**	**3,562**
No. infected at baseline	108	120	21	28	**129**	**148**	533	573	365	252	**898**	**825**	**480**	**834**
Prevalence at baseline (%)	4.7	5.4	1.8	2.8	**3.7**	**4.6**	21.8	23.9	30.1	19.8	**24.5**	**22.5**	**14.3**	**23.4**
No. tested at Year 5	2,440	2,476	1,270	1,172	**3,710**	**3,648**	2,432	2,463	1,189	1,277	**3,621**	**3,740**	**3,689**	**3,724**
No. infected at Year 5	20	7	2	2	**22**	**9**	396	300	443	203	**839**	**503**	**425**	**392**
Prevalence at Year 5 (%)	0.8	0.3	0.2	0.2	**0.6**	**0.2**	16.3	12.2	37.3	15.9	**23.2**	**13.4**	**11.5**	**10.5**
Absolute difference between prevalence at Year 5 and baseline	-3.9	-5.1	-1.6	-2.6	**-3.1**	**-4.4**	-5.5	-11.7	7.2	-3.9	**-1.3**	**-9.1**	**-2.8**	**-12.9**
Relative difference between prevalence at Year 5 and baseline (% change)	-83.0	-94.4	-88.9	-92.9	**-83.8**	**-95.7**	-25.2	-49.0	23.9	-19.7	**-5.3**	**-40.4**	**-19.6**	**-55.1**
Community-level arithmetic mean infection intensity at baseline	1.14	0.73	0.06	0.07	**0.76**	**0.51**	3.14	6.07	6.12	3.22	**4.11**	**5.10**	**1.78**	**5.37**
Community-level arithmetic mean infection intensity at Year 5	0.20	0.07	0.01	0.01	**0.13**	**0.05**	2.05	1.16	6.89	1.84	**3.61**	**1.39**	**1.37**	**2.18**
Egg reduction rate (1-Year 5 intensity/baseline)	82.2%	90.4%	89.7%	90.7%	**82.4%**	**90.4%**	34.8%	80.9%	-12.4%	42.8%	**12.0%**	**72.7%**	**23.3%**	**59.4%**

Individual-level arithmetic mean infection intensity at baseline	1.23	0.80	0.06	0.07	**0.84**	**0.58**	3.21	5.80	5.73	3.22	**4.05**	**4.91**	**1.85**	**5.64**
Individual-level arithmetic mean infection intensity at Year 5	0.21	0.07	0.01	0.01	**0.14**	**0.05**	2.09	1.16	6.72	1.78	**3.61**	**1.37**	**1.37**	**2.20**

Abbreviations: A1, Group A communities randomized to once-a-year MDA in Years 3 and 4; A2, Group A communities randomized to twice a year MDA in Years 3 and 4; B1, Group B communities randomized to once-a-year MDA in Years 3 and 4; B2, Group B communities randomized to twice a year MDA in Years 3 and 4; C, communitywide treatment; H, praziquantel holiday, a year when no praziquantel MDA was provided; S, school-based treatment.
